# ﻿Revisions of the *clavipes* and *pruni* species groups of the genus *Merodon* Meigen, 1803 (Diptera, Syrphidae)

**DOI:** 10.3897/zookeys.1203.118842

**Published:** 2024-05-28

**Authors:** Ante Vujić, Snežana Radenković, Laura Likov, Nataša Kočiš Tubić, Grigory Popov, Ebrahim Gilasian, Mihajla Djan, Marina Janković Milosavljević, Jelena Ačanski

**Affiliations:** 1 University of Novi Sad, Faculty of Sciences, Department of Biology and Ecology, Trg Dositeja Obradovića 2, 21000 Novi Sad, Serbia; 2 I.I. Schmalhausen Institute of Zoology, National Academy of Sciences of Ukraine, Bohdan Khmelnytsky Street 15, UA-01030 Kyiv, Ukraine; 3 Department of Environmental Sciences and Natural Resources, University of Alicante, PO Box. 99, 03080 Alicante, Spain; 4 Insect Taxonomy Research Department, Iranian Research Institute of Plant Protection, Agricultural Research, Education and Extension Organization, Tehran, 19395-1454, Iran; 5 University of Novi Sad, BioSense Institute, Dr Zorana Ðinđića 1, 21000 Novi Sad, Serbia

**Keywords:** Geometric morphometrics, hoverflies, integrative taxonomy, mtDNA COI gene, new species, new synonym

## Abstract

This study focuses on the *avidus–nigritarsis* lineage within the genus *Merodon*, exploring morphological, genetic, and distributional aspects of two related assemblies within this lineage: the *clavipes* and *pruni* species groups. An integrative taxonomic approach was followed to ensure comprehensive species identification and validation, using adult morphology, wing geometric morphometrics, and genetic analysis of the mtDNA COI gene. In the *clavipes* group, seven species were identified, including three new species: *M.aenigmaticus* Vujić, Radenković & Likov, **sp. nov.**, *M.latens* Vujić, Radenković & Likov, **sp. nov.**, and *M.rufofemoris* Vujić, Radenković & Likov, **sp. nov.** In the *pruni* group, our revision revealed a new species, *M.aequalis* Vujić, Radenković & Likov, **sp. nov.**, and the revalidation of *Merodonobscurus* Gil Collado, 1929, **stat. rev.***Merodonpallidus* Macquart, 1842 is redescribed. Diagnoses, identification keys to species, and distribution maps are provided, and neotypes for *Syrphusclavipes* Fabricius, 1781 and *Merodonquadrinotatus* (Sack, 1931) are designated. Additionally, the following new synonyms are proposed: *M.clavipesalbus***syn. nov.**, *M.clavipesater***syn. nov.**, *M.clavipesniger***syn. nov.**, and *M.splendens***syn. nov.** are junior synonyms of *M.clavipes*; and *M.veloxarmeniacus***syn. nov.** and *M.veloxanathema***syn. nov.** are junior synonyms of *M.velox*.

## ﻿Introduction

The genus *Merodon* Meigen, 1803 (tribe Merodontini) is one of the most species-rich hoverfly genera, distributed across the Palaearctic and Afrotropical Regions and comprising 193 described and 41 yet-to-be formally described species (Vujić et al. 2021a). The Mediterranean Basin hosts the highest species diversity (Vujić et al. 2012) with approximately 140 known species (Vujić pers. comm. 21 February 2024), which has been linked to the high diversity of bulb plant species in this region that serve as larval host plants (Ricarte et al. 2008, 2017; Andrić et al. 2014; Preradović et al. 2018). The regions harbouring the greatest species richness are the Iberian, Balkan and especially the Anatolian peninsulas (Vujić et al. 2021a). Asia Minor and Eastern Europe are considered hot spots and regions displaying high endemism levels for the genus (Kaloveloni et al. 2015), as documented by several studies in the Eastern Mediterranean Basin (Vujić et al. 2007, 2011, 2013a, 2015, 2020a, 2020b, 2020c; Ståhls et al. 2009, 2016; Radenković et al. 2011, 2020; Kaloveloni et al. 2015; Ačanski et al. 2016, 2017; Kočiš Tubić et al. 2018; Likov et al. 2020). Central Asia and Pakistan also have numerous endemics with potential significance for the phylogeny of the genus *Merodon* (Vujić et al. 2021a). In contrast, the Afrotropical and Eastern Palaearctic Regions are characterised by having less *Merodon* species (Vujić et al. 2021a).

Vujić et al. (2019) recognised five monophyletic lineages within the genus *Merodon*, i.e., *albifrons*, *aureus*, *avidus*–*nigritarsis*, *desuturinus*, and *natans* lineages, condensing previous studies from Šašić et al. (2016) and Radenković et al. (2018a). Inside the *avidus*–*nigritarsis* lineage, based on the morphological characters and molecular data, ten species groups have been established (*aberrans*, *aurifer*, *avidus*, *clavipes*, *fulcratus*, *italicus*, *nigritarsis*, *pruni*, *serrulatus*, and *tarsatus* groups) together with eight individual taxa without grouping affinities (*M.auronitens* Hurkmans, 1993, *M.caudatus* Sack, 1913, *M.clunipes* Sack, 1913, *M.crassifemoris* Paramonov, 1925, *M.eumerusi*Vujić et al. 2019, *M.hirtus* Sack, 1932, *M.murinus* Sack, 1913, and *M.ottomanus* Hurkmans, 1993) (Likov et al. 2020; Vujić et al. 2021a). Some of these groups were recently revised, such as the *aurifer* (Vujić et al. 2021b), *avidus* (Likov et al. 2020), *nigritarsis* (Vujić et al. 2013a; Likov et al. 2020), *serrulatus* (Vujić et al. 2020b), *aberrans* (Vujić et al. 2022), and *tarsatus* species group (Vujić et al. 2023).

Hurkmans (1988) defined the *clavipes* species group of *Merodon* based on a single apomorphy, i.e., the structure of the anterior surstylar lobe, and he assigned several representatives: *M.aberrans* Egger, 1860, *M.brevis* Paramonov, 1926, *M.clavipes* (Fabricius, 1781), *M.cupreus* Hurkmans, 1988, *M.dzhalitae* Paramonov, 1927, *M.hamifer* Sack, 1913, *M.karadaghensis* Zimina, 1989, *M.lusitanicus* Hurkmans, 1988, *M.quadrinotatus* Sack, 1931, *M.splendens* Hurkmans, 1988, *M.velox* Loew, 1869, and *M.warnckei* Hurkmans, 1988. Nevertheless, Likov et al. (2020) presented this group in a much narrower sense, including large species (15–20 mm) with long body pilosity and a broad metafemur covered with long pile. Likov et al. (2020) only assigned two taxa to the *clavipes* group, namely *M.clavipes* and *M.velox*. Vujić et al. (2021a) mentioned a few additional diagnostic features, such as that the constituent species all: are large and bumble bee-like (15–20 mm) with long body pilosity and a broad metafemur with long pile; have an elongated basoflagellomere; and the male genitalia are well-characterised with large anterior and posterior surstylar lobes. Accordingly, *M.quadrinotatus* and *M.vandergooti* Hurkmans, 1993 were added to the *clavipes* species group.

Hurkmans (1988) established the *pruni* species group based on the structure of the male genitalia, a narrow vertex angle (angle between eyes on male vertex), and the extensive yellow coloration of the abdomen, and he included the nominal species and the variety M.prunivar.obscurus Gil Collado, 1929 as members of this group. In contrast, both Likov et al. (2020) and Vujić et al. (2021a) defined the *pruni* species group based on a completely different set of diagnostic characters: short body pilosity, short basoflagellomere, and the metatrochanter having a distinct calcar. Likov et al. (2020) assigned two species to the *pruni* group, namely *M.pallidus* Macquart, 1842 and *M.pruni* Rossi, 1790, whereas Vujić et al. (2021a) recognised four species in this group, i.e., *M.cupreus* Hurkmans, 1993, *M.pallidus*, *M.pruni*, and one undescribed species from Israel.

Integrative taxonomy, or the use of different sources of information (molecular, morphometric, morphological characters) in the identification and delineation of taxa, has become a widely accepted approach in the taxonomic studies on the genus *Merodon* during the last 15 years. Examples are many for different groupings, like the *avidus* species complex (Popović et al. 2015; Ačanski et al. 2016), and several species groups such as the *ruficornis* (Vujić et al. 2012), *desuturinus* (Vujić et al. 2018), *aureus* (Milankov et al. 2008; Francuski et al. 2011; Šašić et al. 2016; Veselić et al. 2017; Radenković et al. 2018b; Ačanski et al. 2022), *nigritarsis* (Likov et al. 2020), *nanus* (Kočiš Tubić et al. 2018), *serrulatus* (Vujić et al. 2020b), *constans* (Vujić et al. 2020a), *rufus* (Radenković et al. 2020), *natans* (Vujić et al. 2021c), *aberrans* (Vujić et al. 2022), and *tarsatus* species groups (Vujić et al. 2023).

The objectives of the present study are: 1) to review the *clavipes* and *pruni* species group; 2) to define morphological characters for both groups and their constituent species; 3) to study the type material of the species of both groups to resolve nomenclatural issues and to propose appropriate synonyms; 4) to use an integrative taxonomic approach involving molecular and geometric morphometric tools to describe the hidden taxonomic complexity of the taxa in both groups; 5) to describe the new taxa within these groups; 6) to provide identification keys and distributional maps for the species of both groups.

## ﻿Materials and methods

### ﻿Morphological study

In total 947 specimens of the *clavipes* species group and 722 specimens of the *pruni* species group were studied. The examined material belongs to the following institutions and private collections:

**BA coll.** - Barendregt Aat collection, the Netherlands; **BM coll.** – Bartak Miroslav Collection, Czech Republic; **CWM coll.** – de Courcy Williams Michael collection, Greece; **DD coll.** – Doczkal Dieter collection, Germany; **DJ coll.** – Dils Jos collection, Belgium; **EMIT** – Entomological Museum of Isparta, Isparta, Turkey; **FSUNS** – Faculty of Sciences, Department of Biology and Ecology, University of Novi Sad, Novi Sad, Serbia; **GLAHM** – Hunterian Zoology Museum, University of Glasgow, Glasgow, UK; **HM coll.** – Hauser Martin collection, USA; **HMIM** – Hayk Mirzayans Insect Museum, Insect Taxonomy Research Department, Iranian Research Institute of Plant Protection, Tehran, Iran; **IRSNB** – Institut royal des Sciences naturelles de Belgique, Brussels, Belgium; **IZY** – Institute of Zoology, Scientific Center of Zoology and Hydroecology, National Academy of Sciences of the Republic of Armenia, Yerevan, Armenia; **LAU** – Musée Zoologique, Lausanne, Switzerland; **LMR coll.** – Lyszkowski M. Richard collection, Bridge of Allan, UK; **LSF** – Museo Zoologico La Specola, Firenze, Italy; **LT coll.** – Lebard Thomas collection, France; **MAegean** – The Melissotheque of the Aegean, University of the Aegean, Mytilene, Greece; **MNCN** – Museo Nacional de Ciencias Naturales, Madrid, Spain; **MNHN** – Musee National d’Histoire Naturelle, Paris, France; **MZH** – Finnish Museum of Natural History, University of Helsinki, Helsinki, Finland; **MZLS** – Natural History Museum, Zoological Section La Specola, Florence, Italy; **MZLU** – Museum of Zoology Lund University, Lund, Sweden; **NHMB** – Natural History Museum Belgrade, Belgrade, Serbia; **NHMUK** – Natural History Museum, London, UK; **NHMW** – Naturhistorisches Museum Wien, Vienna, Austria; **NMPC** – National Museum Prague, Prague, Czech Republic; **NMS** – National Museum of Scotland, Edinburgh, UK; **PMCG** – Natural History Museum of Montenegro, Podgorica, Montenegro; **RMNH** – Naturalis Biodiversity Center, Leiden, the Netherlands; **SA coll.** – Ssymank Axel collection, Germany; **SD coll.** – Sommaggio Daniele collection, Italy; **SJ coll.** – van Steenis Jeroen collection, the Netherlands; **SIZK** – I. I. Schmalhausen Institute of Zoology, National Academy of Sciences of Ukraine, Kyiv, Ukraine; **SJH coll.** – Stuke Jens-Hermann collection, Germany; **SJM coll.** – Smart J. Malcolm collection, UK; **SZMN** – The Siberian Zoological Museum of the Institute of Systematics and Ecology of Animal Siberian Branch of the Russian Academy of Sciences, Novo Sibirisk, Russia; **TAU** – Tel Aviv University, Tel Aviv, Israel; **THM** – Tullie House Museum & Art Gallery, Carlisle, UK; **TJM coll.** – Taylor J. Mike collection, UK; **USNM** – The Department of Entomology, of the National Museum of Natural History, Smithsonian Institution, Washington, DC, USA; **VWG coll.** – Van de Weyer Guy collection, Belgium; **WK coll.** – Watt Kenneth collection, Aberdeen, UK; **WML** – World Museum Liverpool, Liverpool, UK; **ZFMK** – Museum Koenig, LIB, Bonn, Germany; **ZHMB** – Zoologisches Museum of the Humboldt University, Berlin, Germany; **ZIS** – Zoological Institute and Museum, Sofia, Bulgaria; **ZMBH** – National Museum of Bosnia and Herzegovina, Sarajevo, Bosnia and Herzegovina; **ZMUC** – Zoological Museum, Natural History Museum of Denmark, University of Copenhagen, Copenhagen, Denmark.

The terminology adopted in the morphological descriptions follows Thompson (1999), except terms according to male genitalia which follows Marcos-García et al. (2007), “fasciate maculae” follows Vujić et al. (2021a), and the term “fossette” follows Doczkal and Pape (2009).

In order to study the male genitalia, dry specimens were relaxed in a humidity chamber, and the genitalia were separated from the rest of the specimen using an entomological pin. Genitalia were cleared by boiling them individually in tubes of 10% KOH solution for a few minutes. This process was followed by brief immersion in acetic acid to neutralise the KOH, followed by immersion in ethanol to remove the acid. Genitalia were stored in microvials containing glycerol.

Nikon SMZ18 binocular microscope was used for morphological examination and drawing, while photographs were made using Nikon Digital Sight 10 digital camera. Afterwards, the photographs were stacked in CombineZ software (Hadley 2006). Measurements were taken with an eyepiece graticule or micrometer.

The distribution maps were generated with the mapping software ArcGIS v. 10.3 (ESRI 2014).

### ﻿Molecular study

Genomic DNA of 47 hoverfly specimens belonging to the *clavipes* species group (27 specimens) and *pruni* species group (20 specimens) was obtained for the present study. DNA was extracted from meso- and metalegs using the SDS extraction protocol described by Chen et al. (2010). DNA vouchers of the specimens are deposited at the Department of Biology and Ecology, Faculty of Sciences, University of Novi Sad (FSUNS). Two fragments (the 3′-end and 5′-end) of the mitochondrial COI gene were amplified using C1-J-2183 and TL2-N-3014 primer pair (Simon et al. 1994) and LCO-1490 and HCO-2198 primer pair (Folmer et al. 1994), respectively. The PCR reactions were carried out according to Kočiš Tubić et al. (2018). Amplification products were enzymatically purified using Exonuclease I and Shrimp Alkaline Phosphatase enzymes (ThermoScientific, Lithuania) according to the manufacturer’s instructions and then commercially sequenced in the forward direction by the Macrogen EZ–Seq service (Macrogen Europe, Amsterdam, Netherlands).

Chromatograms of sequences produced for this study were edited for base-calling errors using BioEdit v. 7.2.5. (Hall 1999) and adjusted manually. Additional sequences of species representing the main *Merodon* lineages following Vujić et al. (2021a), as well as sequences of *Platynochaetusmacquarti* Loew, 1862 and *Eumerusgrandis* Meigen, 1822 species serving as outgroups, were retrieved from GenBank and joined to the sequence dataset. The details and GenBank accession numbers of all analysed species and outgroups are presented in Supplementary information (Suppl. material 1). The COI gene sequences of all analysed samples were aligned by the Clustal W algorithm (Thompson et al. 1994) implemented in BioEdit 7.2.5. (Hall 1999). The sequence matrix of concatenated indel-free 3′-end and 5′-end COI gene fragments was used for the construction of two trees: Maximum Parsimony (MP) and Maximum Likelihood (ML). The MP analysis was performed in NONA (Goloboff 1999), spawned with the aid of ASADO, v. 1.85 (Nixon 2008), using the heuristic search algorithm (settings: mult*1000, hold/100, max trees 100000, TBR branch swapping). The ML tree was constructed using MEGA 7.0 software (Kumar et al. 2016) under the general time-reversible evolutionary model (Nei and Kumar 2000) using a discrete Gamma distribution with five rate categories and by assuming that a certain fraction of sites is evolutionarily invariable (GTR+G+I). Nodal support values were estimated using nonparametric bootstrapping with 1000 replicates for both (MP and ML) trees. The trees were rooted on *Platynochaetusmacquarti*.

### ﻿Geometric morphometrics

Landmark-based geometric morphometric analysis of wing shape was conducted on 87 male specimens of the following species: *Merodonclavipes* (*n* = 23); *M.latens* sp. nov. (*n* = 10); *M.obscurus* stat. rev. (*n* = 9); and *M.pruni* (*n* = 45). Female specimens were not available for the analysis. The right wing of each specimen was removed using a micro-scissors and then mounted in Hoyer’s medium on a microscopic slide. Wings have been archived and labelled with a unique code in FSUNS, together with other data relevant to the specimens. Eleven homologous landmarks that could be reliably identified at vein intersections or terminations were selected using TpsDig 2.05 software (Rohlf 2017a) (Table 1). Generalised least squares Procrustes superimposition on the raw coordinates was conducted in TpsRelw v. 1.68 (Rohlf 2017b) to minimise non-shape variations in wing location, scale and orientation and to superimpose the wings in a common coordinate system (Rohlf and Slice 1990; Zelditch et al. 2004).

**Table 1.** Results from discriminant analysis of wing shape differences among investigated species. Above diagonal *p* values. Below diagonal F values. *p < 0.05, **p < 0.01.

	*M.pruni*	*M.obscurus*	*M.latens* sp. nov.	*M.clavipes*
*M.pruni*		0.000171**	0.000000**	0.000000*
*M.obscurus*	3.35056		0.000000**	0.000000**
*M.latens* sp. nov.	27.44203	16.65196		0.000016**
*M.clavipes*	66.27740	28.59318	4.03195	

We performed two separate analyses. First, we assessed wing shape variation among species. Second, we quantified phenotypic differences among geographically-defined groups of specimens (herein treated as populations). Specimens from Italy, Cyprus and France were not included in our population level analysis due to respective small sample sizes, which may interfere with the statistical analysis.

To explore wing-shape variation among the species and populations, we employed discriminant function (DA) and canonical variate (CVA) analyses on a partial warp scores TpsRelw v. 1.68 (Rohlf 2017b). A Gaussian naïve Bayes classifier was also used to delimit species boundaries based on wing shape variation without a priori-defined groups. Phenetic relationships among the species and populations were characterised using an unweighted pair group method with arithmetic mean cluster analysis (UPGMA) based on squared Mahalanobis distances computed from the DA. Superimposed outline drawings produced in MorphoJ v. 2.0 (Klingenberg 2011) were used to visualise differences in wing shape between species pairs. All statistical analyses were performed in Statistica for Windows v. 13 (TIBCO Software Inc. 2018).

## ﻿Results

### ﻿Taxonomic account

***Merodonclavipes* species group**

**Figure 1.**
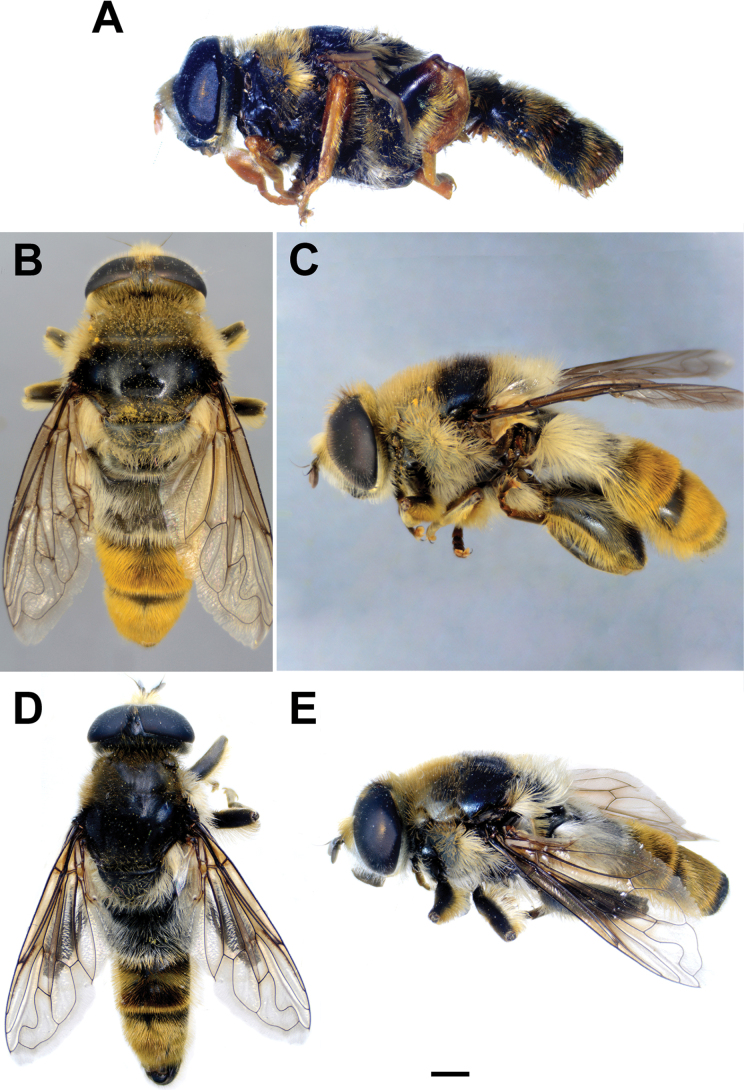
Body of male **A***M.aenigmaticus* sp. nov. **B–C***M.clavipes***D, E***M.latens* sp. nov. **B, D** dorsal view **A, C, E** lateral view. Scale bar: 2 mm.

**Figure 2.**
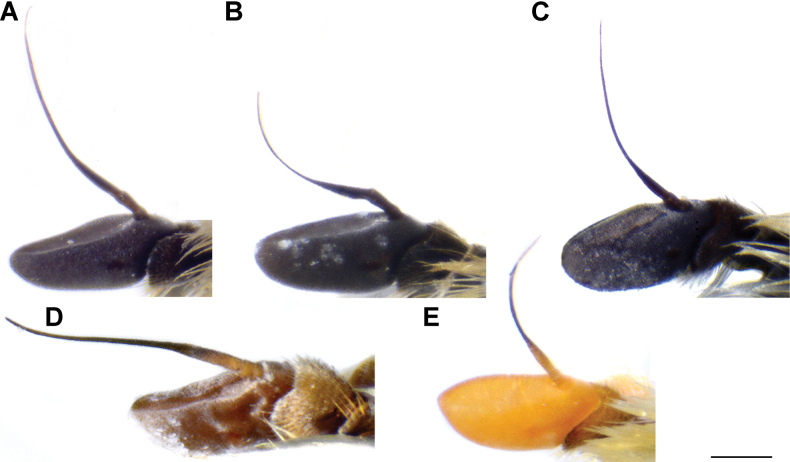
Basoflagellomere of male, lateral view **A***M.clavipes***B***M.latens* sp. nov. **C***M.quadrinotatus***D***M.rufofemoris* sp. nov. **E***M.vandergooti.* Scale bar: 0.5 mm.

**Figure 3.**
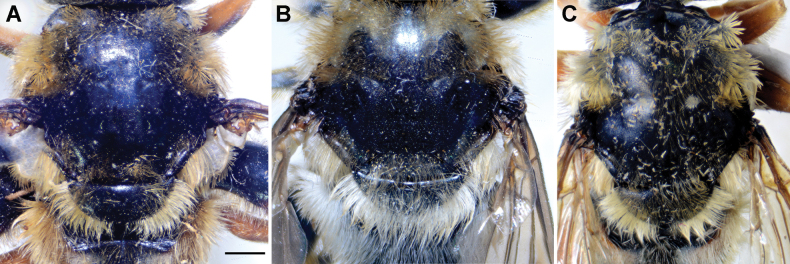
Thorax of male, dorsal view **A***M.aenigmaticus* sp. nov. **B***M.latens* sp. nov. **C***M.rufofemoris* sp. nov. Scale bar: 1 mm.

**Figure 4.**
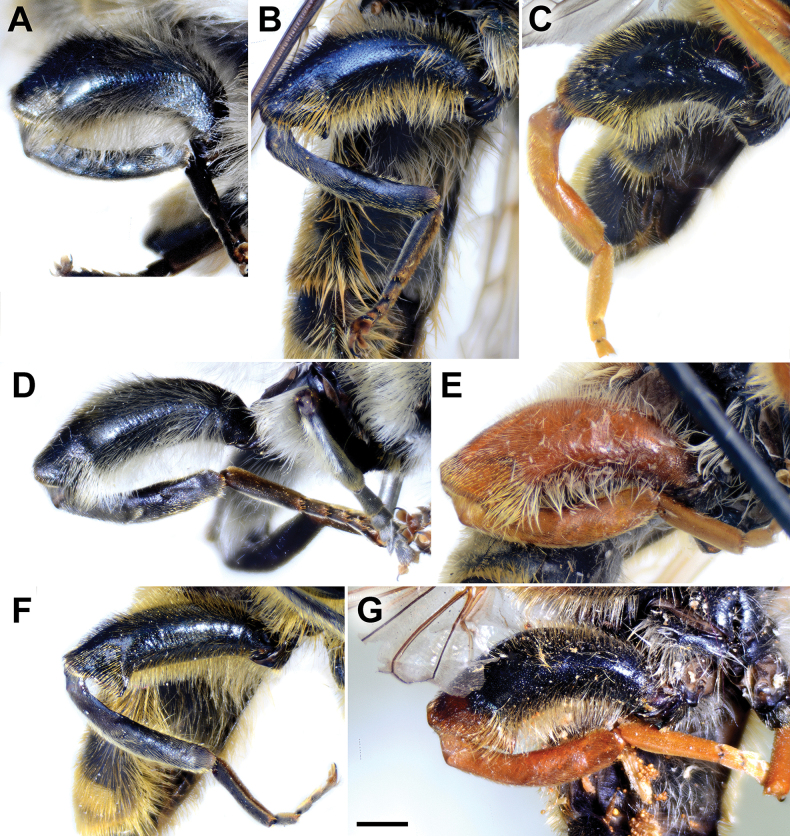
Metaleg of male, lateral view **A***M.clavipes***B***M.latens* sp. nov. **C***M.vandergooti***D***M.quadrinotatus***E***M.rufofemoris* sp. nov. **F***M.velox***G***M.aenigmaticus* sp. nov. Scale bar: 1 mm.

**Figure 5.**
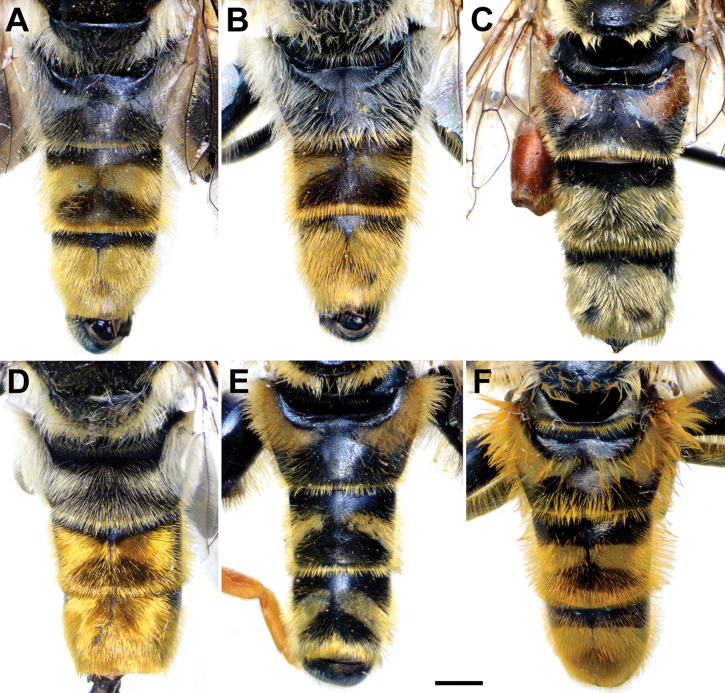
Abdomen of male, dorsal view **A***M.clavipes***B***M.latens* sp. nov. **C***M.rufofemoris* sp. nov. **D***M.quadrinotatus***E***M.vandergooti***F***M.velox.* Scale bar: 2 mm.

**Figure 6.**
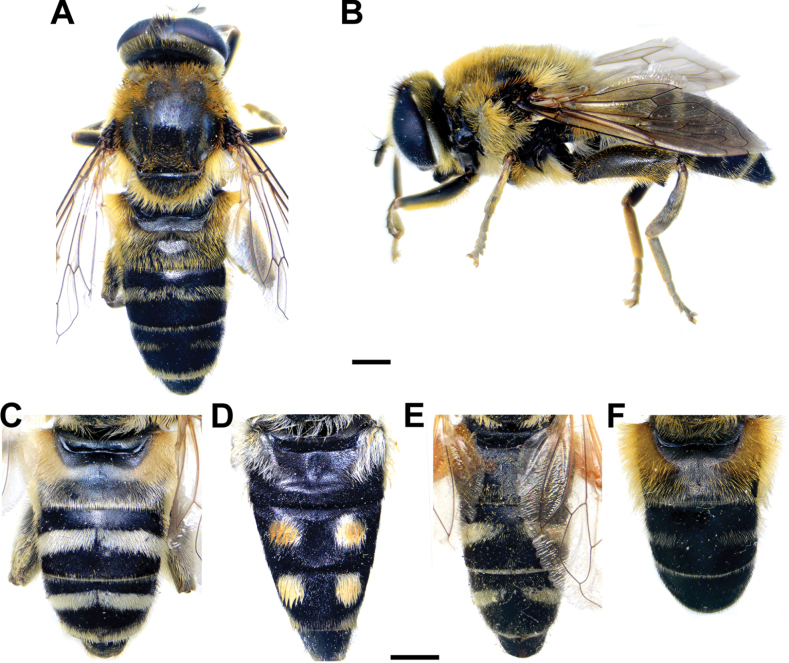
**A, B***M.latens* sp. nov. **C***M.clavipes***D***M.quadrinotatus***E***M.vandergooti***F***M.velox*. **A, B** body of female **C–F** abdomen of female. **A, C–F** dorsal view **B** lateral view. Scale bar: 2 mm.

**Figure 7.**
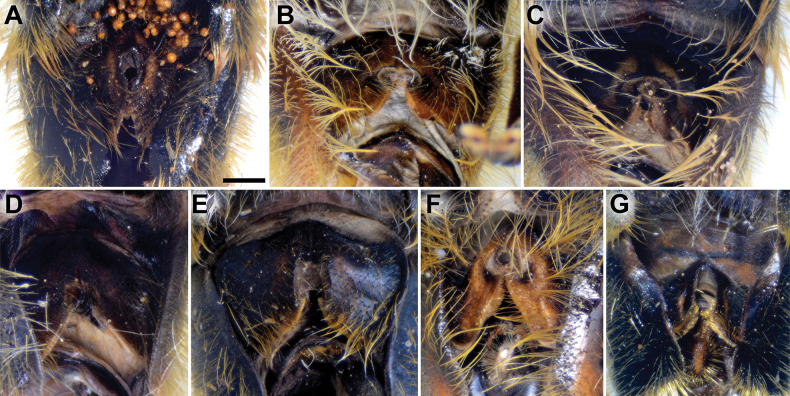
Sternum 4 of male, dorsal view **A***M.aenigmaticus* sp. nov. **B***M.clavipes***C***M.latens* sp. nov. **D***M.quadrinotatus***E***M.rufofemoris* sp. nov. **F***M.vandergooti***G***M.velox*. Scale bar: 1 mm.

**Figure 8.**
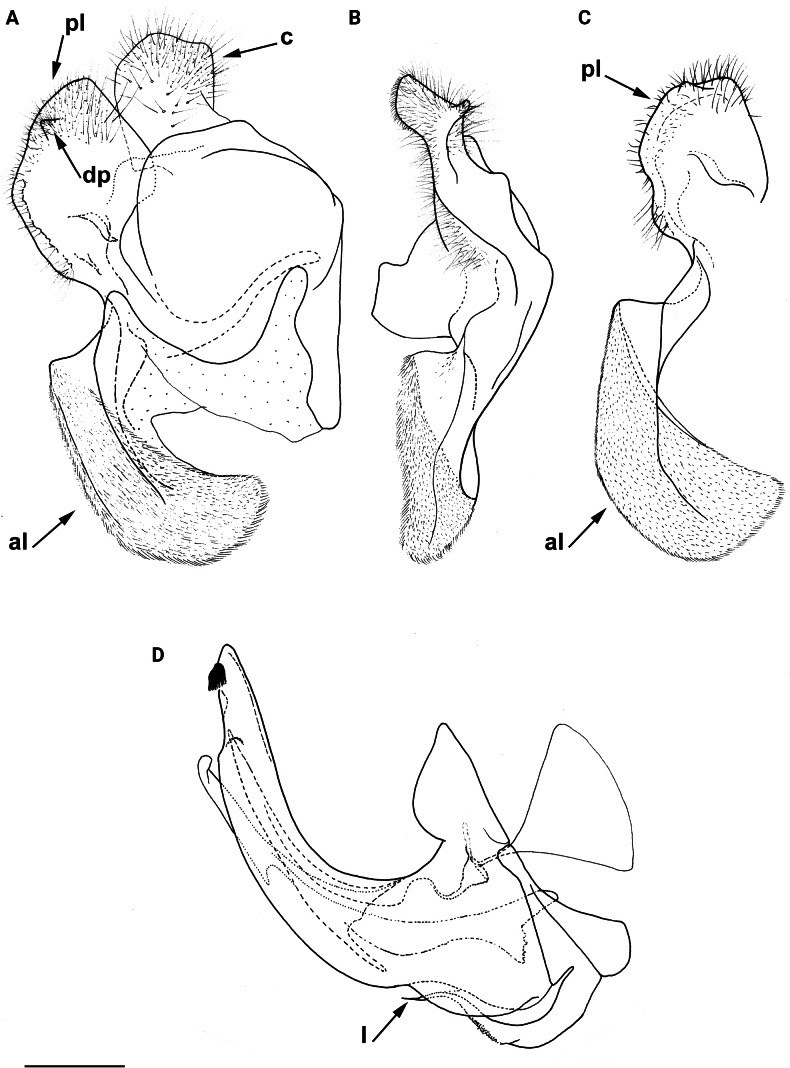
Male genitalia **A, B, D***M.clavipes***C***M.latens* sp. nov. **A–C** epandrium **D** hypandrium. **A, C, D** lateral view **B** ventral view. Abbreviations: al-anterior surstylar lobe, c-cercus, l-lingula, pl-posterior surstylar lobe. Scale bar: 0.5 mm.

**Diagnosis.** The *clavipes* species group belongs to *M.avidus–nigritarsis* lineage, characterised by the mesocoxa without long pile on the posterior section. This group includes large bumble bee-like species (15–20 mm), usually with long body pilosity on thorax, femora and abdomen (Fig. 1B, C); basoflagellomere elongated, > 2× longer than wide (as in Fig. 2); scutum without or with very weak and narrow pollinose vittae (as in Fig. 3) and fascia of completely black or intermixed yellow and black pile between wing bases (as in Fig. 1A, C, E); metatrochanter in male angular (as in Fig. 4F); metafemur broad, covered with very long pile, especially ventrally (Fig. 4); terga black in male, except for the male of *M.rufofemoris* sp. nov. that has tergum 2 with reddish lateral maculae; terga black in females of all species with reddish lateral maculae on tergum 2; terga usually covered with stripes of pile in different combinations of colours (white, yellow or black) (Fig. 5); terga 2–4 with a pair of distinct whitish grey pollinose fasciate maculae (Fig. 6); sternum 4 in male with medial, circular or triangular incision on posterior margin (Fig. 7). Male genitalia: surstylus with well-defined and large anterior and posterior lobes (as in Fig. 8A: al, pl); anterior surstylar lobe large, elongated and sickle-like (Fig. 8A: al); posterior surstylar lobe more or less rectangular (Fig. 8A: pl), in some species with an apicolateral bulge; cercus rectangular (Fig. 8A: c); hypandrium sickle-shaped; lingula distinct, with tapering tip, in some species peak-like (Fig. 8D: l).

The *clavipes* group comprises six species presented here, distributed in the Mediterranean Region and more to the east up to Iran.

#### ﻿Subgroups

**Figure 9.**
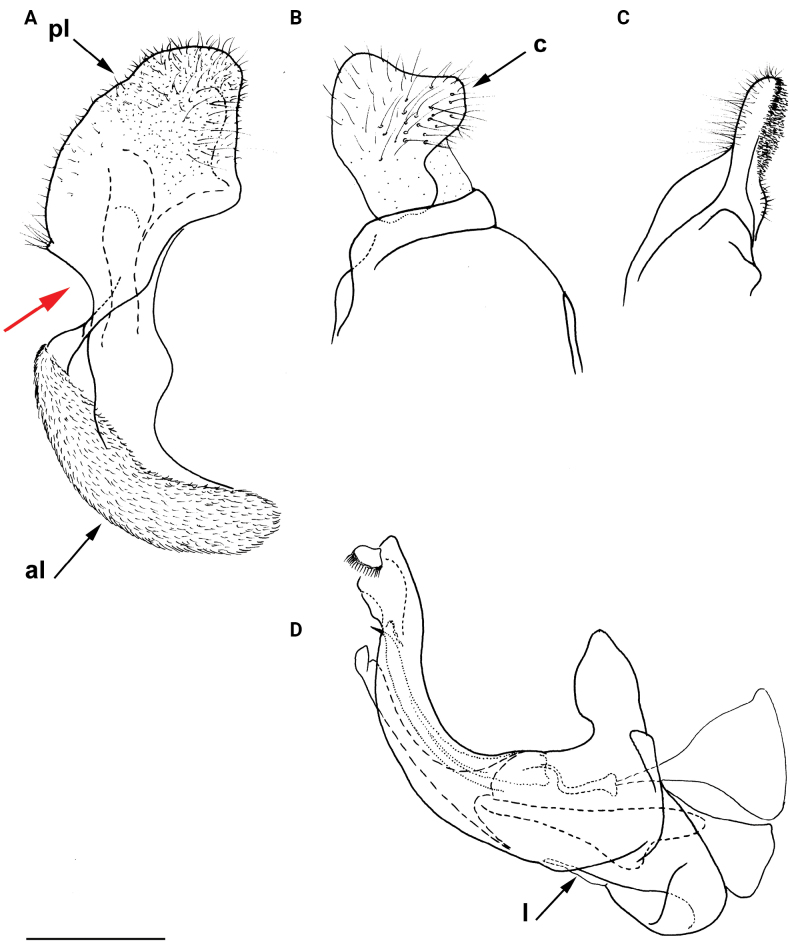
Male genitalia *M.aenigmaticus* sp. nov. **A** surstylar lobe **B** cercus **C** posterior surstylar lobe **D** hypandrium. Rounded posterior surstylar lobe marked with red arrow. **A, B, D** lateral view **C** ventral view. Abbreviations: al-anterior surstylar lobe, c-cercus, l-lingula, pl-posterior surstylar lobe. Scale bar: 0.5 mm.

The *clavipes* species group of *Merodon* contains two subgroups based on the structure of male genitalia, colour of legs and basoflagellomere, and pilosity of posterior margin of scutellum. The *vandergooti* subgroup is characterised by completely or partly orange-yellowish tibiae, tarsi and femora, with bright orange-yellow basoflagellomere, the posterior margin of scutellum without long pile medially (as in Figs 2D, E, 3A, C, 4C, E, G), and posterior surstylar lobe without dorsal prominence (as in Figs 9A: pl, 11A: pl, 10A: pl). This subgroup includes *M.vandergooti* and two species described here, *M.aenigmaticus* sp. nov. and *M.rufofemoris* sp. nov. The other subgroup is the *clavipes* subgroup, whose members have black to dark brown legs and basoflagellomere, and pilosity on posterior margin of scutellum not interrupted medially (as in Figs 2A, 3B, 4A), and the posterior surstylar lobe has a dorsal prominence (as in Fig. 8A: dp, pl, C: pl). This subgroup contains three previously known species, *M.clavipes*, *M.quadrinotatus*, *M.velox* and one species described here, *Merodonlatens* sp. nov.

##### ﻿ Merodon aenigmaticus

Taxon classificationAnimaliaDipteraSyrphidae

Vujić, Radenković & Likov sp. nov.

7213A751-C558-5A71-BC29-313125550CD3

https://zoobank.org/A5E500DF-C71A-4127-966E-25B7B759C2F0

Figs 1A , 3A , 4G , 7A , 9 , 12C

###### Type material examined.

***Holotype*.** Male in MNHN. The specimen had no label or information about its origin. FSUNS ID 04325.

###### Diagnosis

**Figure 10.**
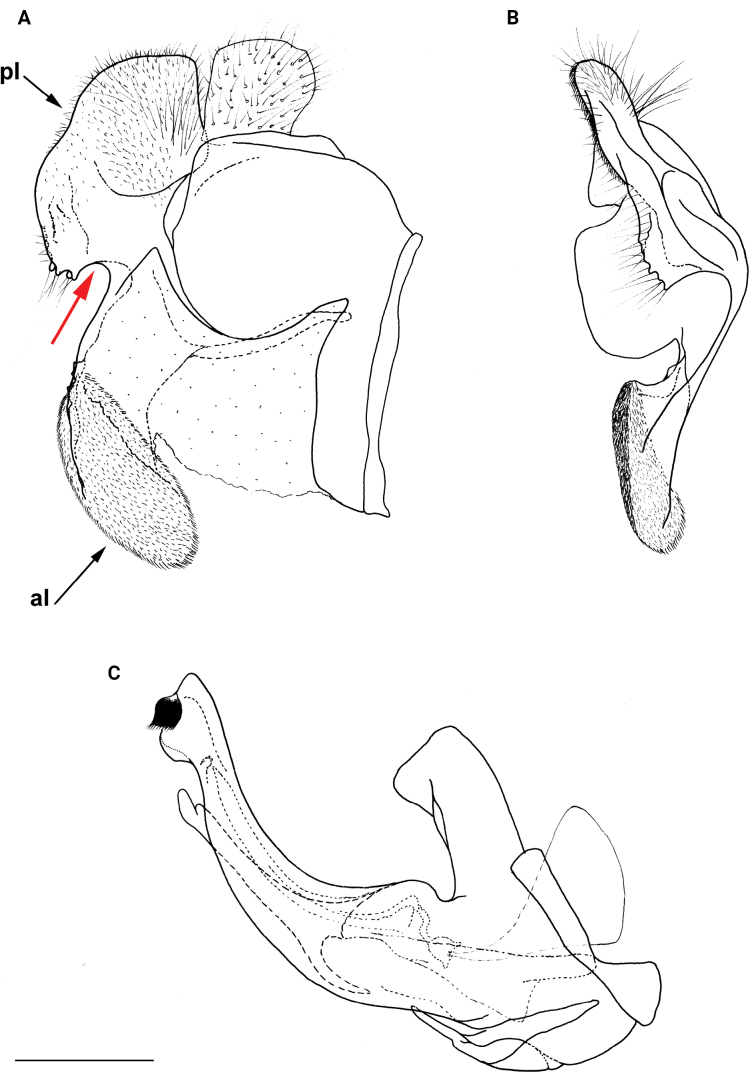
Male genitalia *M.vandergooti***A, B** epandrium, **C** hypandrium. **A, C** lateral view B ventral view. Strongly angulated ventral part of posterior surstylar lobe marked with red arrow. Abbreviations: al-anterior surstylar lobe, pl-posterior surstylar lobe. Scale bar: 0.5 mm.

**Figure 11.**
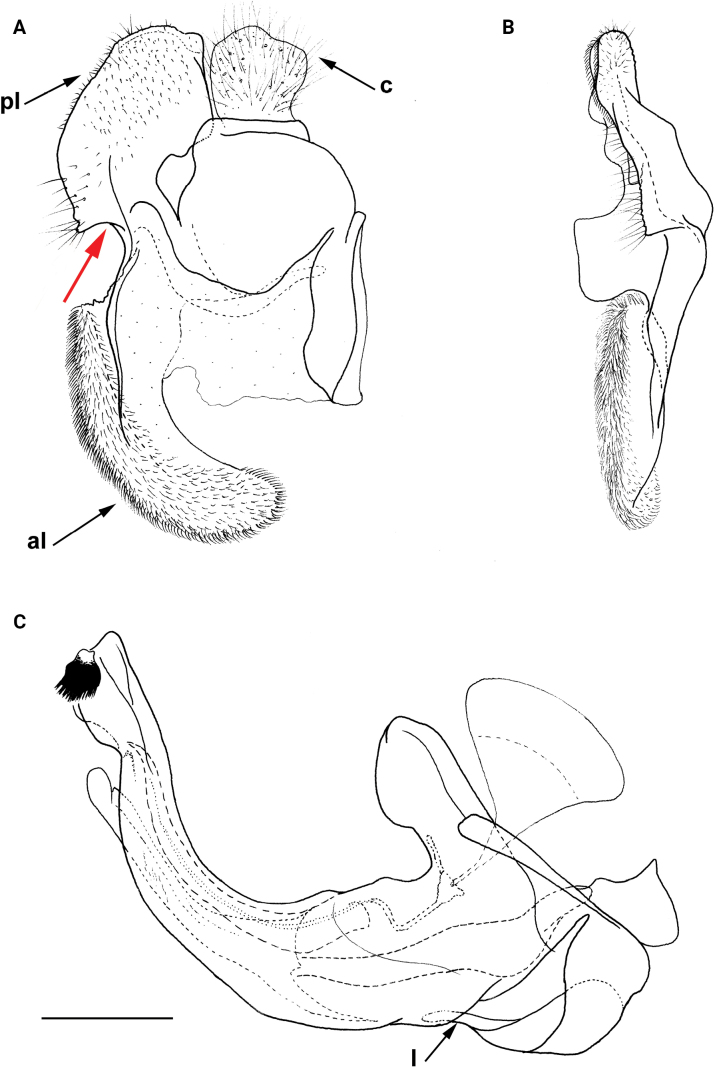
Male genitalia *M.rufofemoris* sp. nov. **A, B** epandrium **C** hypandrium **A, C** lateral view **B** ventral view. Strongly angulated ventral part of posterior surstylar lobe marked with red arrow. Abbreviations: al-anterior surstylar lobe, c-cercus, l-lingula, pl-posterior surstylar lobe. Scale bar: 0.5 mm.

**(only male known).** Similar to *Merodonvandergooti* (Fig. 4C) from which differs with less broad metafemur (in *M.aenigmaticus* sp. nov. is ~ 3.5×, while in *M.vandergooti* is ~ 2.5× longer than wide) (Fig. 4G), less curved metafemur and metatibia (Fig. 4G), and quite rounded posterior surstylar lobe (Fig. 9A: pl, marked with red arrow), while posterior surstylar lobe is strongly angulated ventrally in *M.vandergooti* (Fig. 10A: pl, marked with red arrow). It differs from *M.rufofemoris* sp. nov. by partly black femora (Fig. 4G) (orange-yellow in *M.rufofemoris* sp. nov.; Fig. 4E), and quite rounded posterior surstylar lobe (Fig. 9A: pl) (strongly angulate ventrally in *M.rufofemoris* sp. nov.; Fig. 11A: pl, marked with red arrow).

###### Description.

**Figure 12.**
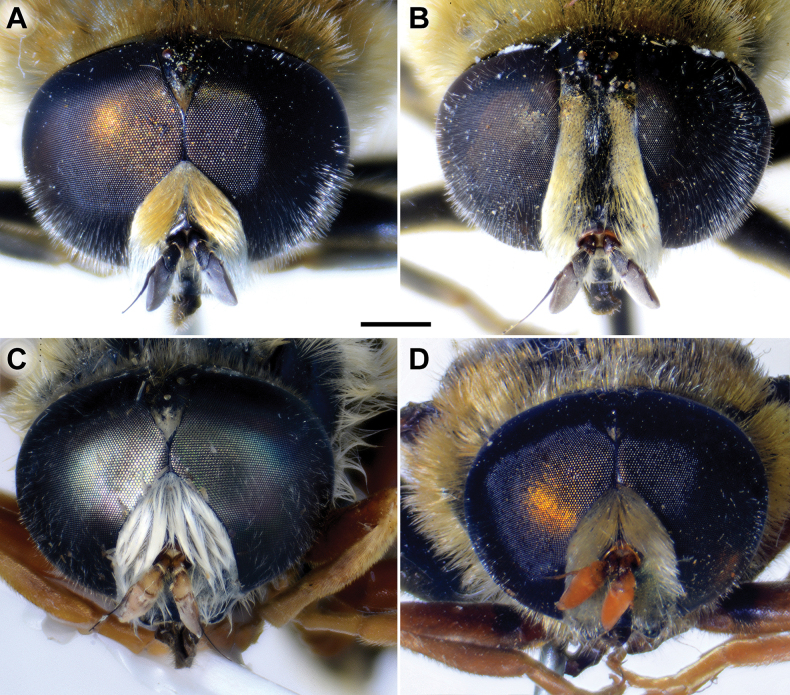
Head, frontal view **A, B***M.latens* sp. nov. **C***M.aenigmaticus* sp. nov. **D***M.rufofemoris* sp. nov. **A, C–D** male **B** female. Scale bar: 1 mm.

Male. Head. Basoflagellomere orange-yellow (Fig. 12C), elongated, ~ 2× longer than wide, and ~ 2.2× longer than pedicel, convex dorsally; fossette dorsolateral; arista reddish to brown and thickened at basal third; arista ~ 1.5× longer than basoflagellomere; face and frons black with whitish pollinosity, while face covered with dense whitish pilosity; pile on frons dense, greyish white; oral margin small, black, sparsely pollinose; lunula shining black to brown, bare; eye contiguity ~ 12 facets long; vertical triangle isosceles, black, shiny, except grey pollinose anterior corner, covered with greyish white pilosity; ocellar triangle equilateral; occiput with a grey-yellow pile, densely covered with grey pollinosity along eyes; eyes covered with short, whitish grey pile (Fig. 12C).

Thorax. Scutum and scutellum black with bronze lustre, covered with short, reddish yellow pile; pilosity between wing bases mostly black; scutum with indistinct pollinose vittae; posterior margin of scutellum with very long reddish yellow to whitish pilosity, reduced medially (Fig. 3A); posterodorsal part of anterior anepisternum, posterior anepisternum (except anteroventral angle), anterior anepimeron, dorsomedial anepimeron, and posterodorsal and anteroventral parts of katepisternum with long, dense greyish white pile; wings mostly covered with microtrichia; wing veins yellowish to brown; calypteres whitish yellow; halteres yellowish; legs reddish yellow, except black basal half of pro- and mesofemora, and basal 4/5 of metafemur; metafemur broad, covered with long, whitish yellow pilosity (Fig. 4G).

Abdomen. Elongated (Fig. 1A), ~ 1.3× longer than mesonotum; terga black, except lateral sides of tergum 2 with reddish yellow maculae; terga 2–4 with broad, distinct silver-grey pollinose fasciate maculae interrupted medially; pile on terga reddish yellow to whitish; sterna black, covered with whitish grey pile; posterior margin of sternum 4 with characteristic posteromedial incision (Fig. 7A).

Male genitalia (Fig. 9). Anterior surstylar lobe large, elongated (up to 3× longer than wide) and sickle-like (Fig. 9A: al); posterior surstylar lobe rectangular with quite rounded ventral margin (Fig. 9A: pl), ~ 1.5× longer than wide, covered with short pile; cercus rectangular (Fig. 9B: c); hypandrium sickle-shaped, without lateral projections; lingula short and tapering (Fig. 9D: l).

Female. Unknown.

###### Distribution.

Unknown. The species is described based on a male holotype from the MNHN collection lacking any label or information about the origin of the specimen.

###### Etymology.

The name *aenigmaticus* derives from the Latin adjective, meaning ‘enigmatic, like an enigma’, in the masculine form. This term describes the absence of any information related to the holotype, including collecting place, date or collector. Species epithet to be treated as an adjective.

***Merodonclavipes* (Fabricius, 1781)**

*Syrphusclavipes* Fabricius, 1781: 427.

*Muscaclauda* Villers, 1789: 463.

*Muscacurvipes* Gmelin, 1790: 2871.

*Syrphusgravipes* Rossi, 1790: 286.

*Merodoncurvipes* Meigen, 1803: 274.

*Merodonsenilis* Meigen, 1822: 356.

*Merodoncanipilus* Rondani, 1865: 131.

Merodonclavipesvar.alba Paramonov, 1926: 90.

Merodonclavipesvar.atra Paramonov, 1926: 91.

Merodonclavipesvar.niger Paramonov, 1926: 90.

*Merodonclavipesalbus* Peck, 1988: 169 (sic! non Paramonov), syn. nov.

*Merodonclavipesater* Peck, 1988: 169 (sic! non Paramonov), syn. nov.

*Merodonclavipesniger* Peck, 1988: 169 (sic! non Paramonov), syn. nov.

*Merodonsplendens* Hurkmans, 1993: 182, syn. nov.

***Syrphusclavipes* Fabricius, 1781: 427**

**Type locality.** Italy. The original description (Fabricius 1781) was based on an unspecified number of syntypes. The lectotype was designated by Hurkmans (1993: 178): male in Sehestedt and Tonder Lund collection (ZMUC). Unfortunately, the type material was destroyed (AV pers. obs.). Two pins from the type collection possess only labels: [*Syrphusclavipes*] and [P 195-1].

**Neotype (designated here).** Male, Italy, Sicily, 20.vi.1914, leg. Trautmann (ZMUC).

A neotype was designated to clarify the taxonomic status of *Merodonclavipes*. Lectotype was designated by Hurkmans (1993) in his revisionary work on genus *Merodon*, but has been destroyed. Data and description are sufficient to ensure recognition of the specimen designated, and the neotype is consistent with what is known of the former name-bearing type from the original description and latter revision. Neotype belongs to the same country (Italy) cited as the original type locality and it is deposited in the same Museum where lectotype was kept (ZMUC).

***Muscaclauda* Villers, 1789: 463**

**Type locality.** France. Synonymy with *Merodonclavipes* was cited in Peck (1988: 168) and Hurkmans (1993: 178). Type material presumably lost.

***Syrphusgravipes* Rossi, 1790: 286**

**Type locality.** Italy. Synonymy with *Merodonclavipes* was cited in Peck (1988: 168) and Hurkmans (1993: 178). Type material presumably lost.

***Merodonsenilis* Meigen, 1822: 356**

**Type locality.** Italy. Synonymy with *Merodonclavipes* was cited in Peck (1988: 168) and Hurkmans (1993: 178). Lectotype was designated by Hurkmans (1993: 178): female “*senilis*” (NHMW) (not found).

***Merodoncanipilus* Rondani, 1865: 131**

**Type locality.** Italy. Synonymy with *Merodonclavipes* was cited in Peck (1988: 168) and Hurkmans (1993: 178). Lectotype was designated by Hurkmans (1993: 178): male in Rondani collection [52] (LSF) (examined).

**Merodonclavipesvar.alba Paramonov, 1926а: 90**

*Merodonclavipesalbus* Peck, 1988: 169 (sic! non Paramonov), syn. nov.

**Holotype (examined).** Female with labels: white, handwritten, bold ink [N 327]; yellowish, handwritten, pale ink, with bluish typographical frame [Valegotsulovo / d. Balta / g. Odessa / 2.vi.25], 47.566923; 29.9389105, Ukraine; pink, handwritten, pale ink, with double typographical frame [*Merodon* / *clavipes* Fabr. / var. alba ♀ / Typus var. nov.] (SIZK).

**Notes.** This taxon was described from a single female, but the specimen storage location was not indicated (Paramonov 1926a: 90) and, until recently, it was not known (Liepa 1969: 4, 20; Hurkmans 1993: 179 “types of either of the varieties ... are considered to be lost”, 205 “lost”, 206). The original description is based on a single specimen, which is the holotype according to article 73.1.2 ICZN (1999) and it is kept in the SIZK collection (Popov 2011). Type locality: Ukraine. The species name is clearly infrasubspecific (1.3.4, 10.2 ICZN 1999) because, as stated by Paramonov himself, the specimen was collected together with the nominal taxon (45.6.1, 45.6.4 ICZN 1999, also see Lingafelter and Nearns 2013). Therefore, this name is not subject to Code 45.6.4.1 (ICZN 1999). The name was first given subspecies rank in Peck’s Catalogue (1988: 169), i.e., «*M.clavipesalbus* Paramonov» (the original gender ending was incorrect and changed, see Article 34.2, ICZN 1999), according to article 45 (g) (ii) ICZN (1985), now corresponding to 45.6.4 (ICZN 1999) (see 45.6.4.1 of ICZN, 1999). However, this is a violation of article 45 (f) (ii) ICZN (1985), now corresponding to 45.6.1, 45.6.4 (ICZN 1999). According to article 45.5.1 (ICZN 1999), Peck adopts authorship of this species name, so we present it as *Merodonclavipesalbus* Peck, 1988, which is a syn. nov. for *M.clavipes* (Fabricius 1781). Later, Hurkmans (1993: 178) erroneously indicated that Peck (1988: 169) listed the name as a “variety”. He also erroneously indicated that S. Ya. Paramonov published the name in 1927 and that the single specimen is a syntype. He left the ranking “variety” for the name (Hurkmans 1993: 179). Colour varieties of *M.clavipes* have been found in multiple populations of this species, similar to the variations reported for *Merodonequestris* (Conn 1976; Han et al. 2018).

**Merodonclavipesvar.atra Paramonov, 1926а: 91**

*Merodonclavipesater* Peck, 1988: 169 (sic! non Paramonov), syn. nov.

**Notes.** This variety was established without reference to the type material, for the male specimens that were in the possession of P. Sack (Germany, now his collection is conserved in the Naturmuseum Senckenberg, Frankfurt am Main) (Paramonov 1926a: 91). The number of types was not given in the original description and their storage location was not indicated, nor were they discovered subsequently (Liepa 1969: 4, 20). The type locality is also unknown. The types of this variety were also not found in the SIZK Department of Entomology collection (G. Popov, in prep.), where the vast majority of Paramonov’s types are stored. Thus, the types are considered lost, as already indicated by W. Hurkmans (1993: 178, 179, 205).

The name “*atra*” by Paramonov is clearly infrasubspecific (see Articles 1.3.4 and 10.2, ICZN 1999), because S. Paramonov (Paramonov 1926а) placed this variety together with others he described for this species (see Articles 45.6.1 and 45.6.4, ICZN 1999). Moreover, he did not report the type locality (see the same Articles; also see Lingafelter and Nearns 2013). Therefore, this name is not subject to the Code (see Article 45.6, ICZN 1999).

The name was given subspecies rank for the first time (see Article 45.6.4.1, ICZN 1999), «*M.clavipesater* Paramonov» (the original gender ending was incorrect and changed, see Article 34.2, ICZN 1999), in Peck’s Catalogue (1988: 169) according to article 45 (g) (ii) ICZN (1985), now corresponding to Article 45.6.4 (ICZN 1999). However, this is a violation of Article 45 (f) (ii) ICZN (1985), now corresponding to Articles 45.6.1 and 45.6.4 (ICZN 1999). So, according to the Articles 45.5.1 and 50.3.1 (ICZN 1999), L. Peck established her own authorship of this name, and we use subspecies name *ater* Peck, 1988 that we consider to be a new synonym (syn. nov.) for *M.clavipes* (Fabricius, 1781), since according to our data, this colour form has no geographical reference and is inherent to some specimens of the species throughout the range. Colour varieties of *M.clavipes* have been found in multiple populations of this species, similar to variations described for *Merodonequestris* (Conn 1976; Han et al. 2018).

**Merodonclavipesvar.nigra Paramonov, 1926а: 90**

*Merodonclavipesniger* Peck, 1988: 169 (sic! non Paramonov), syn. nov.

**Holotype (examined).** Female with labels: white, handwritten, bold ink [N 328]; yellowish, handwritten, pale ink, with bluish typographical frame [Valegozulovo / d. Balta / g. Odessa / 28.v.25.], 47.566923; 29.9389105, Ukraine; pink, handwritten, pale ink, with double typographical frame [*Merodon* / *clavipes* Fabr. / var. nigra ♀ / Typus. var. nov.] (SIZK).

**Notes.** The situation for variety *niger* is identical to that described above for variety *alba* (see above *clavipes* var. alba Paramonov, 1926). The taxon was described from a single female, but the specimen storage place was not indicated (Paramonov 1926a) and, until recently, it was not known (Liepa 1969; Hurkmans 1993). The original description is based on a single specimen, which is the holotype that is kept in the SIZK collection (Popov 2011). Type locality: Ukraine. This name is clearly infrasubspecific because, as indicated by Paramonov himself, the specimen was collected together with the nominal taxon. Therefore, this name is not subject to Code 45.6.4.1 (ICZN 1999). The name was given subspecific rank for the first time in Peck’s Catalogue (1988), i.e., «*M.clavipesniger* Paramonov» (the original gender ending was incorrect and changed, see Article 34.2, ICZN 1999). Thus, Peck assumes authorship of this name, so we use *Merodonclavipesniger* Peck, 1988, which is a syn. nov. for *M.clavipes* (Fabricius, 1781). Later, Hurkmans (1993) mistakenly indicated that Peck (1988) listed the name as a “variety”, that Paramonov published the name in 1927, and that the single specimen is a syntype. He left the rank variety for the name (Hurkmans 1993). Colour varieties of *M.clavipes* have been found in multiple populations of this species, similar to variations described for *Merodonequestris* (Conn 1976; Han et al. 2018).

##### ﻿ Merodon splendens

Taxon classificationAnimaliaDipteraSyrphidae

Hurkmans, 1993: 182 syn. nov.

538FDA8E-51ED-5321-8E28-4D936B8F7DC6

###### Type locality.

Italy, Sardinia. The original description was based on a male holotype (Hurkmans 1993) from Lausanne Museum (LAU). Holotype (designated by Hurkmans): male, Italy, Sardinia (LAU), [specimen dry pinned]. Original labels: [Sardaigne St. Ussassai 16.v.1977 P. Goeldlin], [Holotype of *Merodonsplendens* Hurkmans]. The holotype is conspecific with *Merodonclavipes* (examined).

###### Diagnosis.

Male: legs black (Fig. 4A); antennae black (Fig. 2A); metafemur extremely broad (~ 2–2.5× longer than wide) and curved basally (Fig. 4A); tergum 3 with a pair of rectangular pollinose fasciate maculae, ending close to lateral margins (Fig. 1C). Female with a pair of reddish lateral maculae on tergum 2 (Fig. 6C). Male genitalia in Fig. 8. Similar to *M.latens* sp. nov. from which differs by a broader metafemur, ~ 2–2.5× longer than wide (Fig. 4A) (~ 3–3.5× in *M.latens* sp. nov.; Fig. 4B), and the posterior surstylar lobe more straight ventrally (Fig. 8A: pl) (more arcuate ventrally in *M.latens* sp. nov.; Fig. 8C: pl).

###### Distribution and biology.

**Figure 13.**
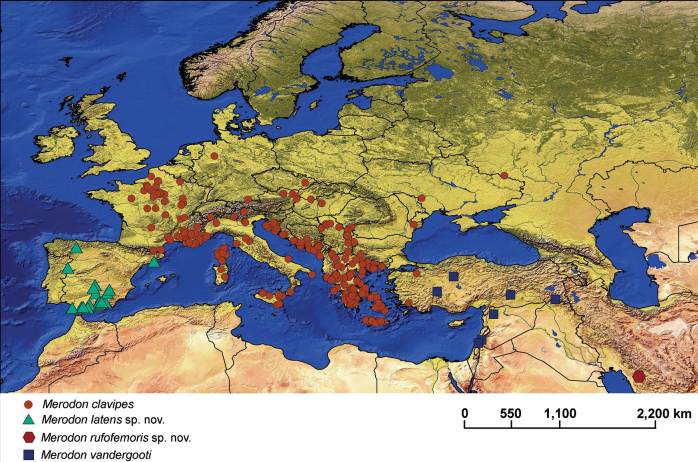
Distribution map of *Merodonclavipes*, *M.latens* sp. nov., *M.rufofemoris* sp. nov. and *M.vandergooti*.

From northern France to the Mediterranean (including Corsica, Sardinia, Sicily and Crete); from Italy through central and southern Europe to Greece, countries of the former Yugoslavia, as well as Albania, Romania, Ukraine (Odesa region, Zakarpattia region), and southern areas of the European parts of Russia and Turkey. Speight (2020) also mentioned North Africa and the Iberian Peninsula as within the species range. Specimens from North Africa were unavailable to us for examination, so we could not confirm if they indeed belong to *Merodonclavipes*. In terms of the Iberian specimens, we assert that they belong to *M.latens* sp. nov. (Fig. 13; Suppl. material 2). The preferred environment of *Merodonclavipes* in the Mediterranean is sparsely-vegetated open ground in semi-arid environments, typified by unimproved stony pasturage and open grassy areas within thermophilous *Quercus* forest (Speight 2020). In the more temperate zone of Europe, the preferred environments are steppe grasslands and open areas near thermophilous forests. In Ukraine, at the northern edge of its range, this species occurs in rocky steppe on the margin of *Quercus* forest (locus typicus of Paramonov’s varieties). Hurkmans (1985) described the territorial behaviour of males and, in Hurkmans (1993), he also noted that females fly close to the soil and through the vegetation. Flowers visited: Umbellifers; *Euphorbia*, *Leontodon* and *Solidago* (Speight 2020). Flight period: March/August depending on climatic zone (in central Europe adults appear during shorter period in early summer, while in southern Europe there can be two generations, spring and summer ones). Developmental stages: undescribed (Speight 2020).

##### ﻿ Merodon latens

Taxon classificationAnimaliaDipteraSyrphidae

Vujić, Radenković & Likov sp. nov.

83AB027E-25BE-5118-A1D4-3B9D07D2046F

https://zoobank.org/6FEF8C5C-26F2-4141-9FC7-DE8BFCE237AC

Figs 1D, E , 2B , 3B , 4B , 5B , 6A, B , 7C , 8C , 12A, B , 13 , 14B , 15 , 17

###### Type material examined.

***Holotype***: Spain • 1 ♂; Sierra Nevada, second valley; 37.102778, -3.455277; 17 Jun. 2014; leg. A. Vujić, S. Radenković, S. Pérez- Bañón; in FSUNS. ***Paratypes*.** Spain • 7 ♂♂, 3 ♀♀; Andalusia, Almijara, Corbijo los Capotes; 36.879, -3.7317; 11 Jun. 2003; leg. D. Doczkal; in DD collection. Spain, Andalusia, Granada, 37.25, -3.25, 29–31 May 1925, leg. Zerny, 1 male in NHMW • 1 ♀; Andalusia, Granada; 1 Jun. 1925; leg. Zerny; in NHMW • 1 ♂, 1 ♀; Andalusia, Puerto de Santa Maria; 36.6401, -6.2596; Apr. 1933; leg. S. Hering; in ZHMB • 1 ♀; Andalusia, Sierra de Baza; 37.422222, -2.851944; 9 Jun. 2003; leg. D. Doczkal; in DD collection • 2 ♂♂; Andalusia, Sierra de Segura, Casas de Carrasco; 38.156666, -2.678333; 7 Jun. 2003; leg. D. Doczkal; in DD collection • 1 ♂; Barcelona; 41.414247, 2.127128; May 1918; leg. H. Teunissen; in RMNH • 1 ♂; Burgos, Espinosa de Cervera; 12 Jun. 1992, 41.897516, -3.467732; leg. M. Hull; in WML • 1 ♂; Castilla la Mancha, Sierra de Alcaraz, Riopar; 38.504722, -2.46; 14 Jun. 2003; leg. D. Doczkal, in DD collection • 3 ♂♂, 2 ♀♀; Ciudad Real, Sierra de Santa Maria, Viso del Marques; 38.966666, -3.9166666; 20 Apr. 1999; leg. M.E. Irwin; in HM collection • 1 ♂; Cortes de la Frontera, way to Grazalema, 36.593904, -5.312444, 6 May 2015; leg. A. Vujić; in FSUNS • 1 ♂, 1 ♀; Cortijo los Capotes, Almijara; 36.878889; -3.731667; 11 Jun 2003; leg. A. Ssymank; in SIZK • 1 ♂; Granada, Rio Lanjaron, near Lanjaron; 36.9437, -3.469431; 28. Apr. 1966; leg. Lyneb. Martin, Langemark; in ZMUC • 3 ♂♂, 2 ♀♀; Granada, Sierra Nevada, near Padul; 37.0833333, -3.1666667; 4 May 1966; leg. Martin; Langemark; in ZMUC • 1 ♂; Grazalema 2, Puerto Alamillo; 36.722683, -5.333724; 8 May 2015; leg. A. Vujić; in FSUNS • 1 ♂; Leon, Mirantes de Luna; 42.841438, -5.861399; 3 Jun. 1987; leg. M.A. Marcos-García; in FSUNS • 1 ♂; Lugros, Sierra Nevada; 37.183056, -3.257778; 18 Jun. 2014, leg. A. Vujić, S. Radenković, S. Pérez-Bañón; in FSUNS • 1 ♂; Malaga, Alhaurin el Grande; 36.633333, -4.683333; 1 May 1979; leg. H. Teunissen; in RMNH • 1 ♂; Malaga, Ronda; 16 Apr. 1955; leg. I.H.H. Yarow; in NHMUK • 1 ♂; Prov. Salamanca, Villar de Ciervo; 40.741661, -6.741098; 24 May 1987; leg. Tschorsnig; in ZFMK • 1 ♂, 1 ♀; Sierra Nevada, first valley; 37.127777, -3.445555; 17 Jun. 2014; leg. A. Vujić, S. Radenković, S. Pérez-Bañón; in FSUNS • 1 ♂; Sierra Nevada, Rio Lanjaron 2; 38.125555, -3.870833; 28 Apr. 2019; leg. A. Vujić, S. Radenković; in FSUNS • 1 ♂, 1 ♀; Sierra Nevada N. P., road to San Jeronimo; 37.240277, -3.48; 17 Jun. 2014, leg. X. Mengual; in ZFMK • 1 ♂; SW Spain, 4 km SE of Antequera; 37.002352, -4.517977; 7 May 1981; leg. A.E. Stubbs; in NHMUK • 1 ♂; Puertollano; 38.697473, -4.090701; in MNHN.

###### Diagnosis.

**Figure 14.**
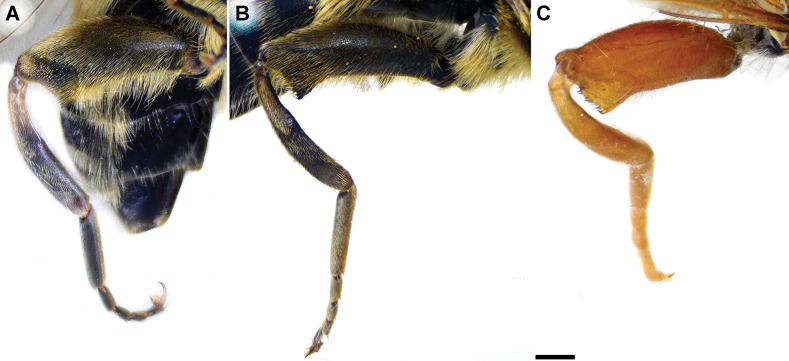
Metaleg of female, lateral view **A***M.clavipes***B***M.latens* sp. nov. **C***M.vandergooti*. Scale bar: 1 mm.

**Figure 15.**
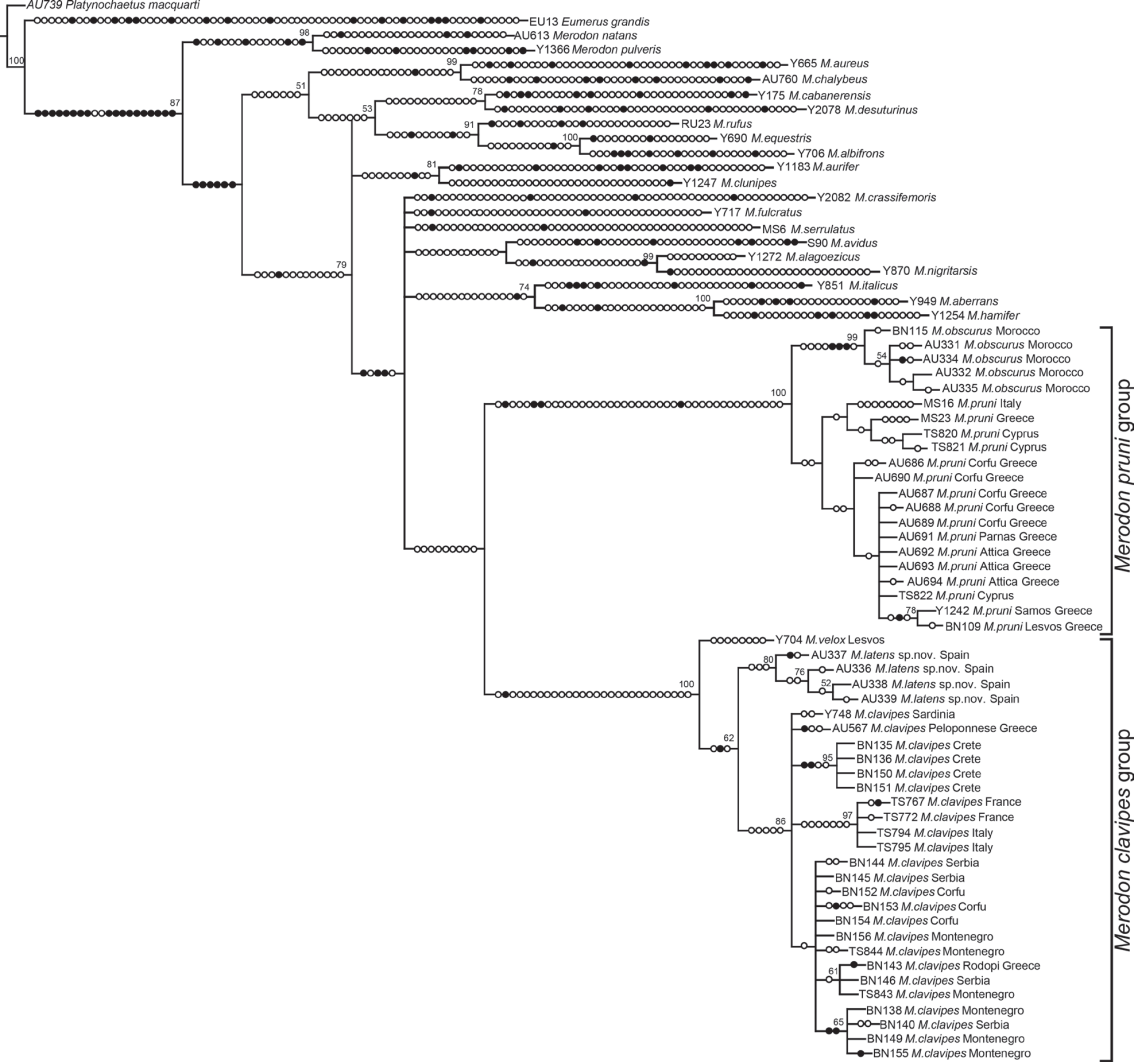
Maximum Parsimony strict consensus tree based on nine equally parsimonious trees, length 1443 steps, consistency index (CI) 38, retention index (RI) 75. Filled circles represent non-homoplasious changes and open circles are homoplasious changes. Bootstrap supports are depicted near nodes (≥ 50).

**Figure 16.**
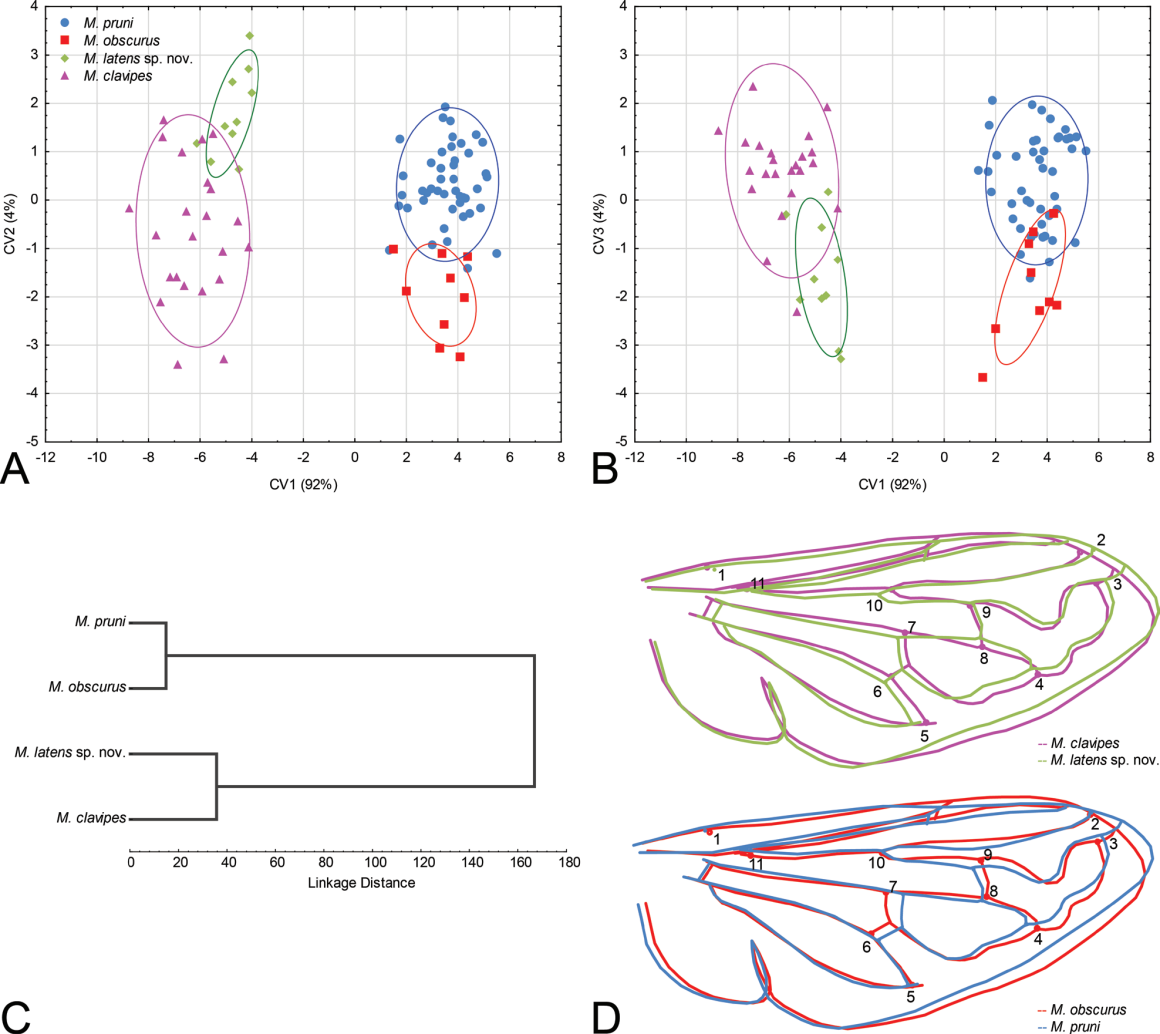
Geometric morphometric analysis of the wing shape in males of *Merodonclavipes*, *M.latens* sp. nov., *M.obscurus*, and *M.pruni***A** Position of male specimens in the space defined by CV1 and CV2 axes **B** Position of male specimens in the space defined by CV1 and CV3 axes **C**UPGMA phenogram constructed using squared Mahalanobis distances of wing shape **D** Drawings showing differences in wing shape for each species pair; differences between the species were exaggerated 5-fold to make them more discernible.

**Figure 17.**
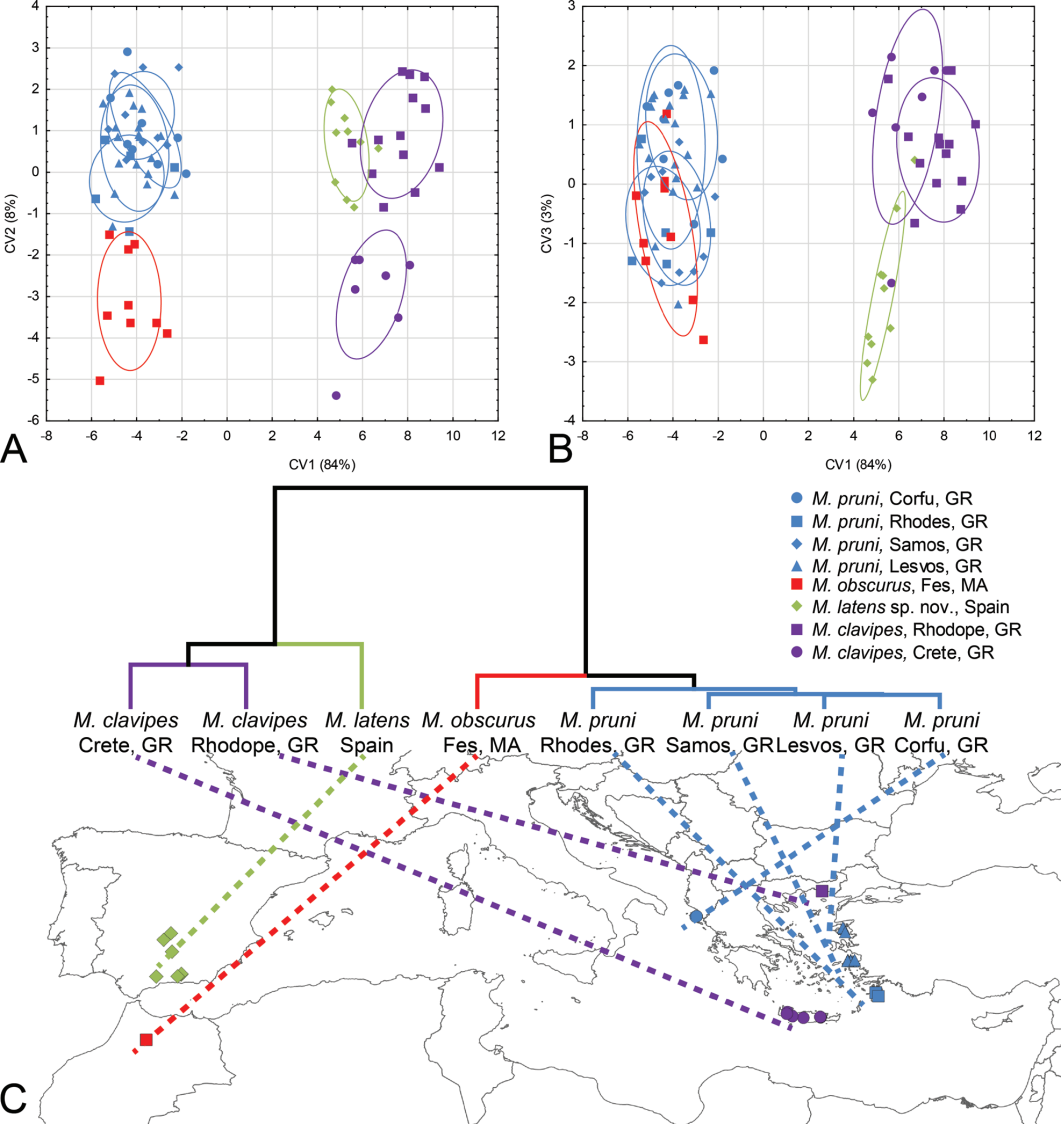
Wing shape differences among populations of *Merodonclavipes*, *M.latens* sp. nov., *M.obscurus* and *M.pruni***A** Scatter plot of individual scores of CV1 and CV2 **B** Scatter plot of individual scores of CV1 and CV3 **C**UPGMA phenogram constructed using squared Mahalanobis distances of wing shape plotted on the map of Mediterranean basin showing the distribution of populations used in the analysis.

Similar to *Merodonclavipes* from which differs by the less broad metafemur of the male (from lateral view ~ 4× longer than wide; Fig. 4B) (< 3× longer than wide in *M.clavipes*; Fig. 4A), less curved metafemur basally (strongly curved in *M.clavipes*; Fig. 4A), and ventral pilosity on metafemur < 2× longer than dorsolateral (Fig. 4B) (while > 2× longer in *M.clavipes*; Fig. 4A). Male genitalia are very similar to *M.clavipes* (Fig. 8A), with the single difference in the shape of surstylus, especially of the posterior surstylar lobe: more arcuate ventrally in *M.latens* sp. nov. (Fig. 8C: pl), and more or less straight in *M.clavipes* (Fig. 8A: pl). Female of *M.latens* sp. nov. has less dense ventral pilosity on metafemur, with ventral pile as long as a dorsolateral pile (Fig. 14B), while female of *M.clavipes* has denser and longer ventral pilosity on metafemur (Fig. 13A). Molecular and morphometric data clearly separated these two species (Figs 15, 16, 17 and Suppl. material 3). *Merodonlatens* sp. nov. is an Iberian endemic.

###### Description.

Male. Head. Basoflagellomere dark brown (Fig. 2B), elongated, ~ 2× longer than wide, and ~ 2.3× longer than pedicel, convex dorsally; fossette dorsolateral; arista brown and thickened at basal third; arista ~ 1.3× longer than basoflagellomere (Fig. 2B); face and frons black, with whitish grey pollinosity; face covered with dense whitish pilosity; pile on frons dense, grey-yellow; oral margin small, black, sparsely pollinose; lunula shining black to brown, bare; eye contiguity ~ 13–15 facets long; vertical triangle isosceles, black, shiny, except grey pollinose anterior corner, covered with both black and yellow pile; ocellar triangle equilateral; occiput with grey-yellow, dense pollinosity; eyes densely covered with whitish grey pile (Fig. 12A).

Thorax. Scutum and scutellum black with bronze lustre, covered with short, greyish yellow pile in anterior half; pilosity between wing bases entirely or mostly black; scutum with indistinct pollinose vittae; transverse suture with two medial pollinose maculae; posterior margin of scutum and all scutellum with long whitish pilosity (Fig. 3B); posterodorsal part of anterior anepisternum, posterior anepisternum (except anteroventral angle), anterior anepimeron, dorsomedial anepimeron, and posterodorsal and anteroventral parts of katepisternum with long, dense, whitish pile; wings mostly covered with microtrichia; wing veins brown to black; calypteres whitish yellow; halteres yellow to brown; legs black; metafemur moderately broad, from lateral view ~ 4× longer than wide, covered with long, whitish, yellow, and black pile (Fig. 4B).

Abdomen. Elongated (Fig. 5B), as long as mesonotum; terga black; terga 3 and 4 with distinct silver-grey pollinose fasciate maculae interrupted medially; pile on terga 1 and 2 whitish, while on terga 3–5 grey-yellow to reddish; sterna black, covered with whitish yellow pile; posterior margin of sternum 4 with characteristic circular posteromedial incision (Fig. 7C).

Male genitalia (Fig. 8C). Anterior surstylar lobe large, elongated and sickle-like (Fig. 8C: al); posterior surstylar lobe rectangular, arcuate ventrally (Fig. 8C: pl).

Female (Fig. 6A, B). Similar to the male except for typical sexual dimorphism and the following characteristics: frons with broad pollinose vittae along eyes, occupying ~ 1/3 of the width of the frons from frontal view (Fig. 12B); scutum between wing bases without black pilosity, only wing basis with few black pile in some specimens (Fig. 6B); metafemur narrower (~ 3.5× longer than wide), with ventral pilosity shorter than in male (Fig. 14B); lateral sides of tergum 2 with reddish yellow maculae (Fig. 6A); terga 3–5 with short adpressed black pilosity medially.

###### Distribution and biology.

The species range is limited to the Iberian Peninsula (Spain) (Fig. 13). It preferentially occurs in open sparsely-vegetated semi-arid environments, typically unimproved stony pasturage and open grassy areas within thermophilous *Quercus* forest. Adult males and females both showed territorial behaviour, flying close to the soil and through the vegetation. Flowers visited by adults are mostly umbellifers and *Euphorbia*. Flight period: April/June. Developmental stages: undescribed.

###### Etymology.

The name *latens* derives from the Latin adjective meaning hidden, secret, not revealed. This term refers to the discovery of Iberian populations, previously cited as *Merodonclavipes*, as distinct species. Species epithet to be treated as an adjective.

##### ﻿ Merodon quadrinotatus

Taxon classificationAnimaliaDipteraSyrphidae

(Sack, 1931)

7E37F932-0334-5761-8539-C190D05A0479

Lampetia quadrinotata Sack, 1931: 324.

###### Type locality.

“Mesopotamia” (Iraq according to Peck 1988). The original description was based on one female (holotype) (Sack 1931). The holotype is considered lost (Hurkmans 1993).

Neotype (designated here): female, Iran, (HMIM), [specimen dry pinned]. Original labels: [IRAN-Fars-Meimand/Firouzabad-Tange riz/N 28 56 00 2670m/E 052 50 07.6/Leg. Gilasian/15.iv.2006], [*Merodonquadrinotatus*/(Sack, 1931)/det. A. Vujić 2019], [Loan Vujic 2007/Gilasian 32] [NEOTYPE of *Merodonquadrinotatus* Sack / designated by Vujić A.]. A neotype for *Lampetiaquadrinotata* is here designated to fix and ensure the universal and consistent interpretation of the name. This designation was based on the good condition of the specimen; a well-preserved female with clearly visible characters which are conspecific with the holotype. This species possesses a unique character, a pair of tear like white pilose maculae on terga 2 and 3, especially distinct in females (Fig. 6D).

###### Notes.

This species was described based on a single female. Here we present the first description for the male.

###### Diagnosis.

**Figure 18.**
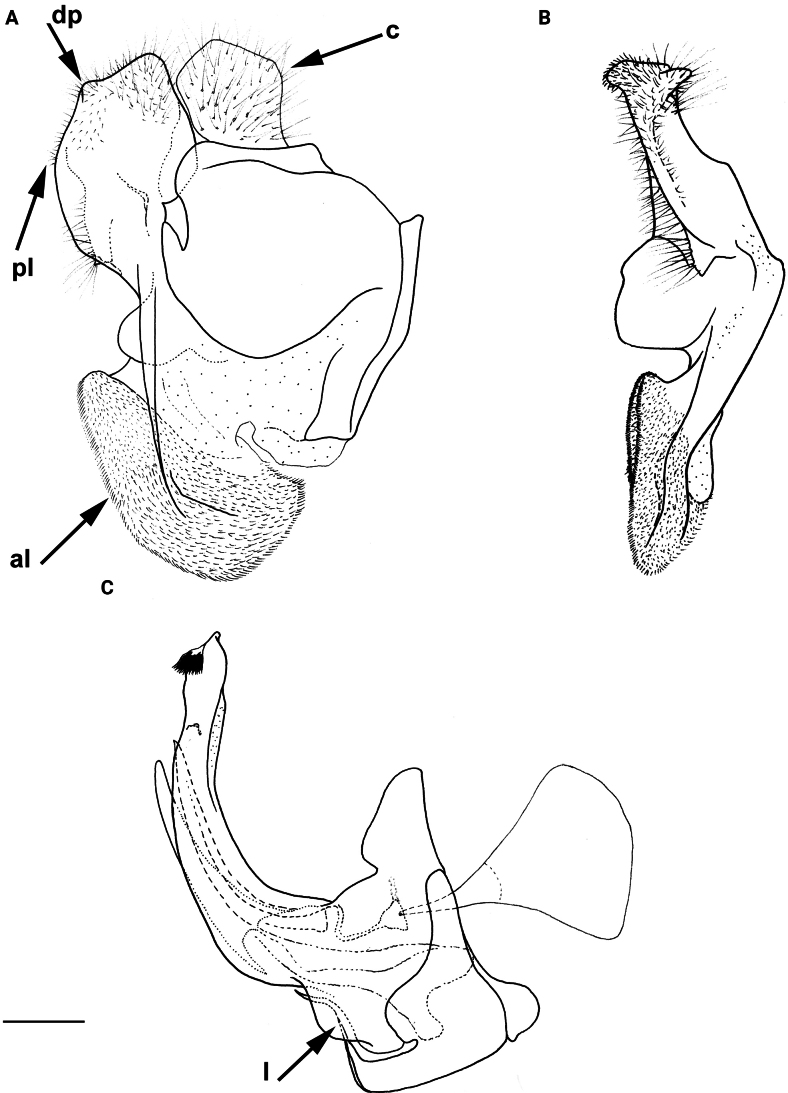
Male genitalia *M.quadrinotatus***A, B** epandrium **C** hypandrium. **A, C** lateral view **B** ventral view. Abbreviations: al-anterior surstylar lobe, c-cercus, dp-dorsal prominence, l-lingula, pl-posterior surstylar lobe. Scale bar: 0.5 mm.

Male similar to *Merodonclavipes* (Figs 4A, 5A) from which differs by the metafemur slightly broad (in *M.quadrinotatus* is 3.75×, while in *M.clavipes* is 2× longer than wide) and less curved basally (Fig. 4D) and by tergum 3 with a pair of tear-like, pollinose fasciate maculae separated from lateral margins (Fig. 5D) (in *M.clavipes* tergum 3 with a pair of rectangular pollinose fasciate maculae, ending close to lateral margins). Female with black terga and very characteristic pairs of pollinose, rounded maculae covered with dense whitish pile on terga 3 and 4 (Fig. 6D); a unique abdominal pattern in *Merodon*. Male genitalia as in Fig. 18.

###### Description.

Male. Head. Basoflagellomere dark-brown (Fig. 2C), elongated, ~ 2× longer than wide, and ~ 1.9× longer than pedicel, convex dorsally; fossette dorsolateral; arista reddish to brown and thickened at basal third; arista ~ 1.5× longer than basoflagellomere; face and frons black, with greyish pollinosity; face covered with dense whitish to yellowish pilosity; pile on frons dense, yellowish; oral margin small, black, not pollinose; lunula shining black to brown, bare; eye contiguity ~ 15 facets long; vertical triangle isosceles, brown-black, shiny, except grey pollinose anterior corner, covered with greyish white and black pilosity; ocellar triangle equilateral; occiput with grey-yellow pile, densely covered with grey pollinosity along eyes; eyes covered with short, whitish grey pile.

Thorax. Scutum black with bronze lustre, covered with greyish yellow pile; pilosity between wing bases mostly black; scutum with indistinct pollinose vittae; scutellum covered with whitish pile; posterior margin of scutellum with very long grey-yellow to whitish pilosity, reduced medially (as on Fig. 3C); posterodorsal part of anterior anepisternum, posterior anepisternum (except anteroventral angle), anterior anepimeron, dorsomedial anepimeron, and posterodorsal and anteroventral parts of katepisternum with long, dense whitish to greyish white pile; wings mostly covered with microtrichia; wing veins yellowish to brown; calypteres whitish; halteres brownish; legs black; metafemur moderate broad, ~ 3.75× longer than wide, covered with long, whitish pilosity (Fig. 4D).

Abdomen. Elongated (Fig. 5D), ~ 1.3× longer than mesonotum; terga black; terga 3 and 4 with a pair of broad, tear-like, distinct silver-grey pollinose fasciate maculae; pile on tergum 2 and lateral sides of terga 3 and 4 grey-yellow to whitish; terga 3 and 4 medially with short, golden-yellow pile (Fig. 5D); sterna black, covered with whitish grey pile; posterior margin of sternum 4 with characteristic posteromedial incision (Fig. 7D).

Male genitalia (Fig. 18). Anterior surstylar lobe short (~ 1.4× longer than wide) and rectangular (Fig. 18A: al); posterior surstylar lobe rectangular, with a dorsal prominence (Fig. 18A: dp); cercus rectangular (Fig. 18A: c); hypandrium sickle-shaped, without lateral projections; lingula short, with tapering narrow tip (Fig. 18C: l).

Female. Similar to the male except for normal sexual dimorphism and the following characteristics: face and frons covered with white pilosity; frons with broad pollinose vittae along eyes and a narrow shiny central stripe; scutum with short erect white pilosity, except for broad fascia of black pile between wing bases; long whitish pilosity on metafemur absent; metafemur covered with short black pilosity and few longer black pile ventrally; terga covered with short black pilosity, except for long white pile on lateral sides of terga 2–4, posterior margin of tergum 4, and pairs of pollinose, rounded maculae covered with dense whitish pile on terga 3 and 4 (Fig. 6D).

###### Distribution and biology.

**Figure 19.**
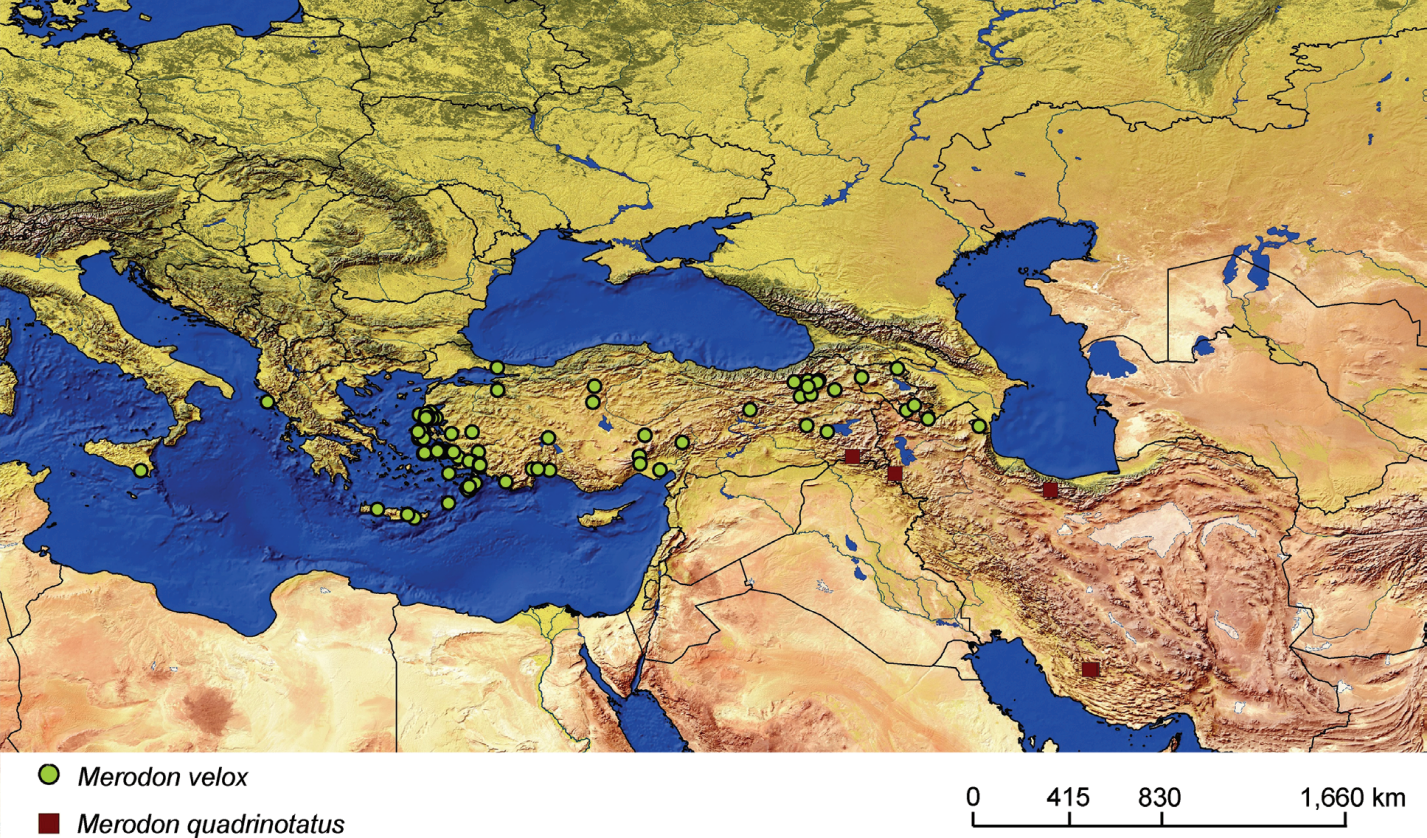
Distribution map of *Merodonvelox* and *M.quadrinotatus*.

The range of this species includes Turkey, Iran and Iraq (Fig. 19; Suppl. material 2). *Merodonquadrinotatus* has been recorded predominantly in Iranian ecoregions, specifically, forest steppe of the Zagros Mountains, Eastern Anatolian montane steppe, and woodlands and forest steppe of Kopet Dag (Kopeh Dagh) (Olson et al. 2001) but also in nearby localities within Iraq and Turkey. The Iranian localities are typified by arid and semi-arid forest ecosystems with *Quercusbrantii* Lindl. as the dominant vegetation type, as well as cold and arid semi-steppe scrubland and grasslands (*Astragalus* spp.) (Azizi Jalilian et al. 2020). The preferred environment is sparsely-vegetated open ground in semi-arid regions, with unimproved stony pasturage and open grassy areas within thermophilous forest being typical. Flight period: April/June. Developmental stages: undescribed.

##### ﻿ Merodon rufofemoris

Taxon classificationAnimaliaDipteraSyrphidae

Vujić, Radenković & Likov sp. nov.

46E4029A-7509-5397-B1CF-71858ACFECF4

https://zoobank.org/85A45AAA-5D78-4914-A8CC-A3E0F8C4DA4A

Figs 2D , 3C , 4E , 5C , 7E , 11 , 12D , 13 , 20A, B

###### Type material examined.

***Holotype***: IRAN • 1 ♂; Fars prov., Dasht-e Ajran; 29.552, 51.942; 5 May 2015; leg. M. Kafka; in BM collection.

###### Diagnosis

**Figure 20.**
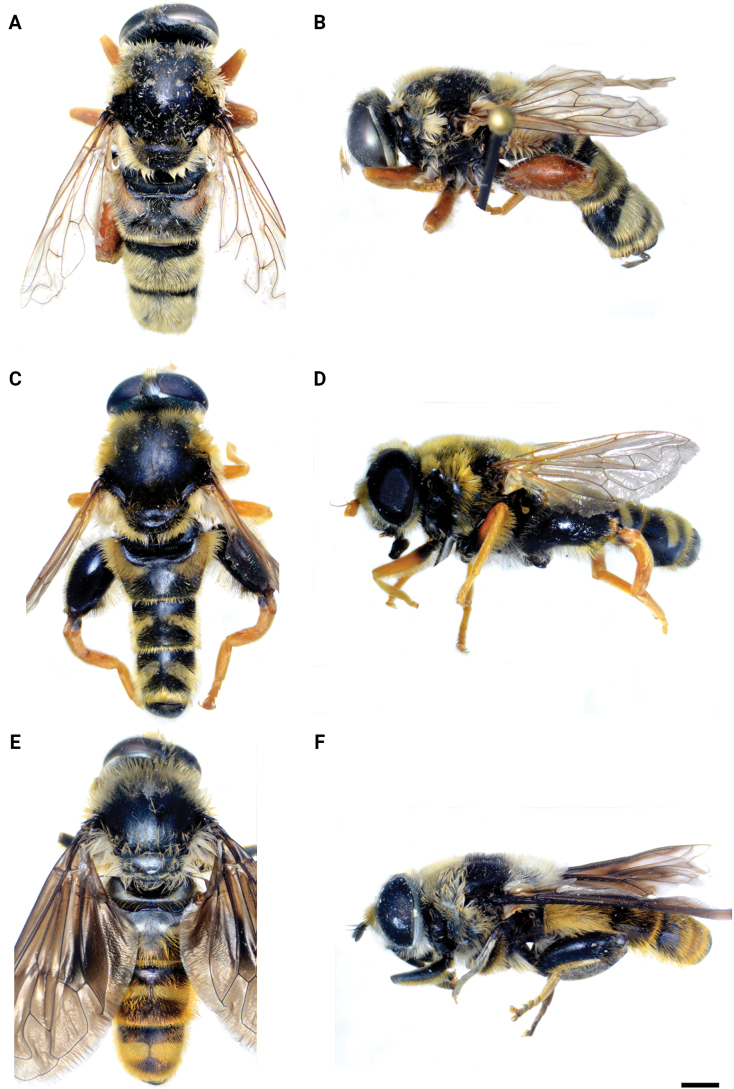
Body of male **A, B***M.rufofemoris* sp. nov. **C–D***M.vandergooti***E, F***M.velox.***A, C, E** dorsal view **B, D, F** lateral view. Scale bar: 1 mm.

**(only male known).** Similar to *Merodonvandergooti* from which differs by all femora completely reddish yellow (Figs 4E, 20B), while in males of *M.vandergooti* pro- and mesofemora are partly orange-yellowish and metafemur is almost completely black (Figs 4C, 20D), a less curved metafemur (Fig. 4E), and an elongated anterior surstylar lobe in *M.rufofemoris* sp. nov. (Fig. 11A: al) (shorter in *M.vandergooti*; Fig. 10A: al). It differs from *M.aenigmaticus* sp. nov. by the reddish yellow femora (Fig. 4E) (partly black in *M.aenigmaticus* sp. nov.; Fig. 4G), and the posterior surstylar lobe angulate ventrally (Fig. 11A: pl) (rounded in *M.aenigmaticus* sp. nov.; Fig. 9A: pl).

###### Description.

Male. Head. Basoflagellomere orange-yellow (Fig. 2D), elongated, ~ 2× longer than wide, and ~ 1.9× longer than pedicel, convex dorsally; fossette dorsolateral; arista reddish to brown and thickened at basal third; arista ~ 1.5× longer than basoflagellomere; face and frons black, with whitish pollinosity; face covered with dense whitish pilosity; pile on frons dense, whitish; oral margin small, black, sparsely pollinose; lunula shining black to brown, bare; eye contiguity ~ 10 facets long; vertical triangle isosceles, black, shiny, except grey pollinose anterior corner, covered with greyish white pilosity; ocellar triangle equilateral; occiput with grey-yellow to reddish pile, densely covered with grey pollinosity along eyes; eyes covered with short, whitish grey pile (Fig. 12D).

Thorax. Scutum and scutellum black with bronze lustre, covered with short, greyish yellow pile; pilosity between wing basis mostly black; scutum with indistinct pollinose vittae; transverse suture with two medial pollinose maculae (Figs 3C, 20A); posterior margin of scutellum with very long grey-yellow to whitish pilosity, reduced medially (Fig. 3C); posterodorsal part of anterior anepisternum, posterior anepisternum (except anteroventral angle), anterior anepimeron, dorsomedial anepimeron, and posterodorsal and anteroventral parts of katepisternum with long, dense greyish white pile; wings mostly covered with microtrichia; wing veins yellowish to brown; calypteres whitish yellow; halteres yellow to white; legs reddish yellow; metafemur broad, ~ 3.5× longer than wide, covered with long, whitish yellow pilosity (Fig. 4E).

Abdomen. Elongated (Fig. 5C), ~ 1.3× longer than mesonotum; terga black, except lateral sides of tergum 2 with reddish yellow maculae; terga 3 and 4 with a pair of broad, distinct silver-grey pollinose fasciate maculae; pile on terga grey-yellow to whitish; sterna black, covered with whitish grey pile; posterior margin of sternum 4 with characteristic posteromedial incision (Fig. 7E).

Male genitalia (Fig. 11). Anterior surstylar lobe large, elongated (~ 3.5× longer than wide) and sickle-like (Fig. 11A: al); posterior surstylar lobe rectangular (Fig. 11A: pl, marked with red arrow); cercus rectangular (Fig. 11A: c); hypandrium sickle-shaped, without lateral projections; lingula short, with tapering but rounded tip (Fig. 11C: l).

Female. Unknown.

###### Distribution and biology.

This species is only found in the Fars Province of Iran (Fig. 13). This Iranian locality lies within the Zagros Mountains forest steppe ecoregion (Olson et al. 2001), representing an arid and semi-arid forest ecosystem with *Quercusbrantii* as the dominant vegetation type (Azizi Jalilian et al. 2020). Flight period: May. Developmental stages: undescribed.

###### Etymology.

The name is derived from the Latin adjective *rufus* (red, reddish) and inflection of the noun femur in genitive singular (*femoris*) and refers to the reddish yellow colour of femora. Species epithet to be treated as an adjective.

##### ﻿ Merodon vandergooti

Taxon classificationAnimaliaDipteraSyrphidae

Hurkmans, 1993

AD4DD646-22DD-55B1-9578-E6A6671AEE1A

Merodon aureotibia Hurkmans, 1993: 203.
Merodon vandergooti Hurkmans, 1993: 188.

###### Type locality.

Turkey, “Hakkari”. The original description was based on a male holotype and ~ 40 male paratypes (all in RMNH) (Hurkmans 1993). Holotype (designated by Hurkmans): male, Turkey, Hakkari (RMNH), [specimen dry pinned]. Original labels: [Turkey, Hakkari, Süvarihalil geçidi, 1250 m W side near Halub Deresi, 13.vi.1984 leg. J. A. W. Lucas], [Holotype of *Merodonvandergooti* Hurkmans] (examined).

***Merodonaureotibia* Hurkmans, 1993: 203**

**Type locality.** Turkey, “Adıyaman”. The original description was based on a female holotype and three female paratypes (all in RMNH) (Hurkmans 1993). Holotype (designated by Hurkmans): female, Turkey, Adıyaman (RMNH), [specimen dry pinned]. Original labels: [Turkey, Adıyaman, Nemrut Dağı, 1.vi.1983, leg. M. Kuhbandner], [Holotype of *Merodonaureotibia* Hurkmans] (examined).

**Notes.***Merodonvandergooti* and *M.aureotibia* were described in the same publication (Hurkmans 1993): *M.vandergooti* from a large number of males and *M.aureotibia* based only on females. Hurkmans (1993) considered *M.vandergooti* is the only member of the *vandergooti* group and *M.aureotibia* as part of the *alagoezicus* group. The type material of the two taxa belongs to the same species, and Vujić et al. (2011) retained *M.vandergooti* (Hurkmans 1993: 188) as the valid name for this species and designated *M.aureotibia* (Hurkmans 1993: 203) as a synonym.

**Diagnosis.** Tibiae and tarsi plus all femora in female (Fig. 14C) while pro- and mesofemora in males partly, orangish yellow (Fig. 20C, D); male metafemur very broad (~ 2.5× longer than wide) and strongly curved, covered with long and dense yellow pile ventrally (Fig. 4C). Male genitalia in Fig. 10.

**Distribution and biology.** The species range includes Israel, Syria and Turkey (Fig. 13; Suppl. material 2). The preferred environment of *Merodonvandergooti* is Eastern Mediterranean conifer-sclerophyllous-broadleaf forests. In Israel, this species has been registered from the Hermon and Meiron mountains where the montane forest is dominated by Quercusinfectoriasubsp.veneris (A. Kern.) Meikle, *Q.libani* G. Olivier, *Juniperusdrupacea* Labill., and Acermonspessulanumsubsp.microphyllum (Boiss.) Bornm., as well as in Mediterranean maquis and semi-steppe bathas (Danin 1988). In Turkey, the species range covers warm temperate grassland and shrubland/woodland (Evrendilek and Gulbeyaz 2008). Flight period: April/July. Developmental stages: undescribed.

***Merodonvelox* Loew, 1869**

*Merodonvelox* Loew, 1869: 253.

Merodonveloxvar.anathema Paramonov, 1926: 149.

Merodonveloxvar.armeniaca Paramonov, 1926: 147.

*Merodonveloxanathemus* Peck, 1988: 175 (sic! non Paramonov), syn. nov.

*Merodonveloxarmeniacus* Peck, 1988: 175 (sic! non Paramonov), syn. nov.

***Merodonvelox* Loew, 1869: 253**

**Type locality.** Turkey, “Smyrna = Izmir” and Greece (Rhodus = Rhodos). The original description (Loew 1869) was based on seven males and an unspecified number of female syntypes from the Vienna collection (RMNH). Lectotype (designated by Hurkmans 1993: 183): male, Greece, Rhodes (NHWM), [specimen dry pinned]. Original label: [Rhodus / Alte Sammlung] (examined).

**Merodonveloxvar.anathemaParamonov 1926b: 149**

*Merodonveloxanathemus* Peck, 1988: 175 (sic! non Paramonov), syn. nov.

**Holotype (examined).** Female with labels: white, handwritten, bold ink [N 340]; printed [mons Takältu / prope Kulp. / 28...V.......13.], = Tekaltı Dağı mountain, near Kulp (Turkey), 38.516667; 41.016667; pink, handwritten, pale ink, with double typographical frame [*Merodon* / *anathema* / n. sp. ♀ Typus / Paramonov d.].

**Notes.** The taxon was described as a “var.” from a single female, which is the holotype according to article 73.1.2 ICZN (1999). Paramonov indicated that the type is kept in his personal collection (Paramonov 1926b: 149). Type locality: Turkey. Later, S. Ya. Paramonov gave the species as “*M.anathema* sp. n.” (Paramonov 1927: 15). Until recently, the type was believed to be lost (Liepa 1969: 4, 20; Hurkmans 1993: 183 “holotype ... not examined, probably lost”, 205 “lost”, 206), but it has since been found at the SIZK (Popov 2011). The name was correctly (see also Lingafelter and Nearns 2013) given a subspecific rank for the first time in Peck’s Catalogue (1988: 175), «*Merodonveloxanathemus* Paramonov», according to 45 (g) (ii) ICZN (1985), now 45.6.4 (ICZN 1999), but the original feminine name *anathema* was incorrectly changed contrary to article 31 (b) (ii) (ICZN 1985), now 31.2.1, 34.2.1 (ICZN 1999). Hurkmans (1993: 184) left the rank variety for the name. The study of the *Merodonvelox* material revealed that character of this subspecies are not outside the limits of species variability in other parts of the species’ range, so we consider *anathema* syn. nov. for *M.velox* Loew, 1869.

**Merodonveloxvar.armeniacaParamonov 1926b: 147**

*Merodonveloxarmeniacus* Peck, 1988: 175 (sic! non Paramonov), syn. nov.

**Lectotype (examined).** Male with labels: white, handwritten, bold ink [N 341]; pale ink [Армения / Эривань / 24.v.24.], = Yerevan (Armenia), 40.166667; 44.516667; pink, handwritten, pale ink, with double typographical frame [*Merodon* / *velox* Lw. ♂ / var. armeniaca / var. nov. / Paramonov det.] (SIZK).

**Paralectotype (examined)**: female with labels: white, handwritten, bold ink [N 342]; pale ink [Армения / Ордубад / 7.VI.24.], = Ordubad (Azerbaijan), 38.908056N 46.027778E; pink, handwritten, pale ink, with double typographical frame [*Merodon* / *velox* Lw ♀ / var. armeniaca / var. nov. / Paramonov det.].

**Notes.** Paramonov indicated that the male types (12 specimens) are kept in two localities, “Typus in meiner Sammlung und im Museum von Armenien” (Paramonov 1926b: 148), with the only female type being kept in his personal collection (ibid.: 149). The exact location of the types was not known, and it was thought that they had possibly been lost (Liepa 1969: 4, 20; Hurkmans 1993: 183 “syntypes ... not examined, probably lost”, 205 “lost”, 206). Two syntypes of 13 have been preserved in SIZK (Popov 2011). It was assumed that some of the syntypes had been preserved at the current IZY (Liepa 1969: 4, 20 “Museum of Natural History of the Armenian SSR, Yerevan”; Hurkmans 1993: 184 “possibly some of the material might be present in the collection of the Museum of Armenia, Erivan”). According to personal communication with Mark G. Kalashyan (Yerevan), a single specimen of *Merodonvelox* is deposited in the IZY collection and was examined by S. Ya. Paramonov, hosting two labels, [Armenia, prope Beuk-Vedi, 1.vi.1926, A. Schelk.] = Vedi, Armenia, 39.910556; 44.727778, and [*Merodonvelox* Lw., ♂, Paramonov d.]. This specimen is not the type. The name was correctly given a subspecific rank for the first time in Peck’s Catalogue (1988: 175) (see also Lingafelter and Nearns 2013), «*Merodonveloxarmeniacus* Paramonov», according to article 45 (g) (ii) ICZN (1985), now corresponding to 45.6.4 (ICZN, 1999). Hurkmans (1993: 184) left the rank of variety for the name. According to article 74 ICZN (1999), we designate the male as the lectotype and the female as the paralectotype. Type locality: Armenia (76.2 ICZN 1999). Paramonov later mentioned this name (Paramonov 1927: 15), but erroneously indicated the wrong year of collection for the types (1925). In fact, 1924 is specified in the original description and indicated on the type labels. The study of the *M.velox* material revealed that characters of this subspecies are not outside the limits of the species variability in other parts of the species range, so we consider *armeniacus* syn. nov. for *M.velox* Loew, 1869.

**Figure 21.**
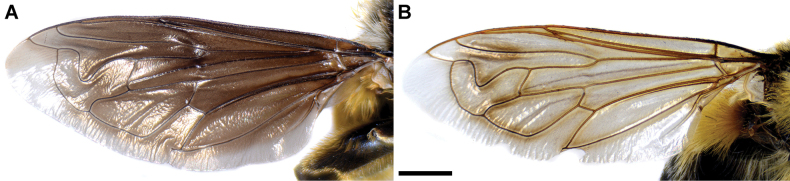
Wing of *Merodonvelox*, dorsal view **A** male **B** female. Scale bar: 1 mm.

**Figure 22.**
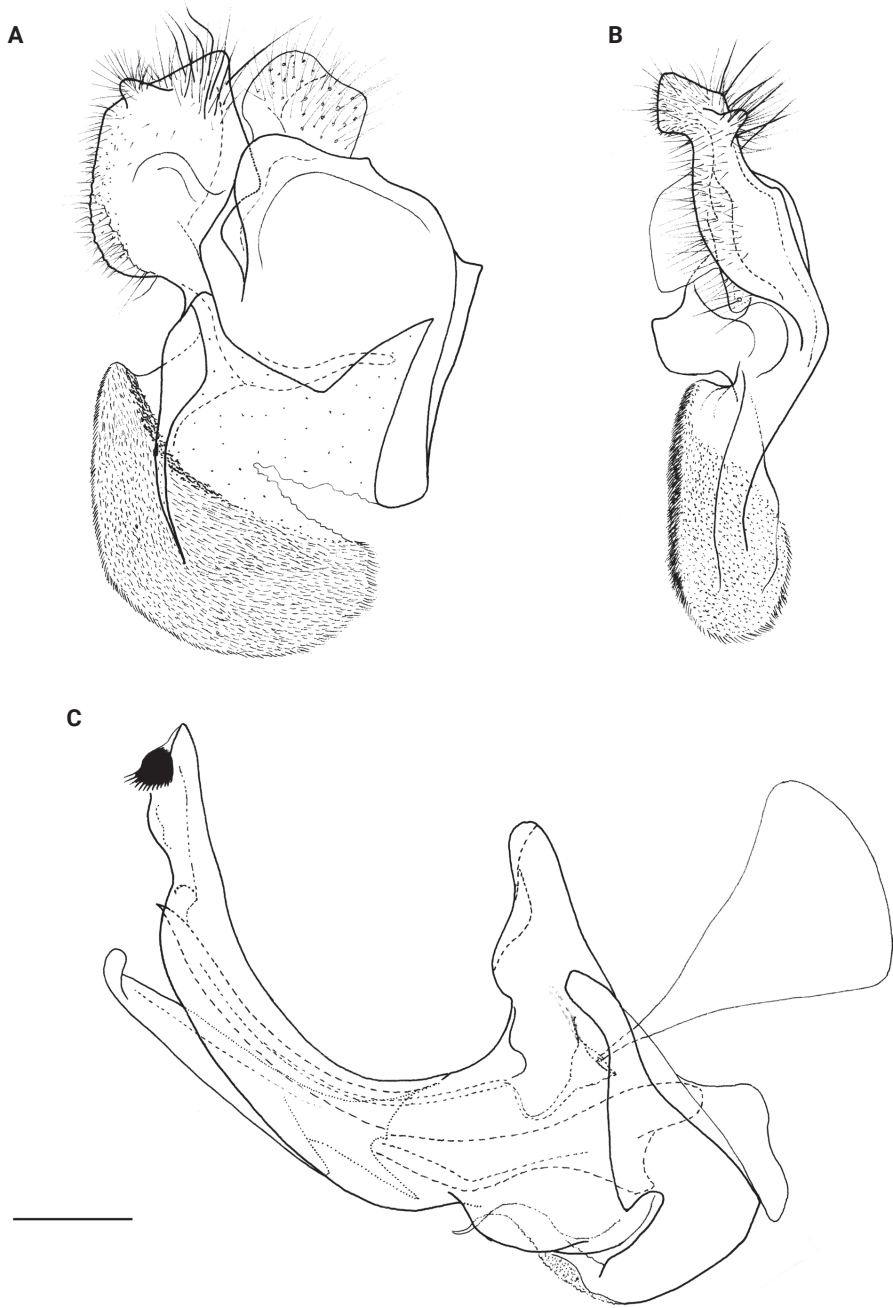
Male genitalia *M.velox***A, B** epandrium **C** hypandrium. **A, C** lateral view **B** ventral view. Scale bar: 0.5 mm.

**Diagnosis.** Male: wings brown-black except extreme apical part (Figs 20E, F, 21A); female: wing in basal half with yellow, while in apical half with brown veins; wing covered along veins with dark brown microtrichia (Fig. 21B). Male genitalia as in Fig. 22. Similar to *Merodonclavipes* and *M.latens* sp. nov. from which male differs by brown-black wing (hyaline wing in *M.clavipes* and *M.latens* sp. nov.) and a narrower metafemur (Fig. 4F), < 2× broader than the metatibia (metafemur is > 2× broader than the metatibia in *M.clavipes* (Fig. 4A) and *M.latens* sp. nov. (Fig. 4B)); female differs by wing covered along veins with dark brown microtrichia (Fig. 21B), clear in *M.clavipes* and *M.latens* sp. nov.

**Distribution and biology.** The species range includes Armenia, Azerbaijan, Georgia, Greece, Italy, and Turkey. Hurkmans (1993) also lists Yugoslavia, but those records could not be confirmed (Fig. 20; Suppl. material 2). The preferred environment of *Merodonvelox* is forest or open ground, typically thinly-vegetated and stony semi-arid areas, unimproved grasslands, and open areas in *Abies* forest, as well as *Castanea* forest (Speight 2020). This species apparently resembles a small *Xylocopa* in the field and continues to fly at temperatures above 35 °C. Males are strongly territorial, and both sexes fly low and fast through ground vegetation (Hurkmans and Hayat 1997). The species has been found drinking at the edge of a small stream in the evening on a hot day (Reemer and Smit 2007). Flowers visited: umbellifers; *Euphorbia* (Zimina 1960; Hurkmans and Hayat 1997). Flight period: March/September. Developmental stages: not described (Speight 2020).

### ﻿Key for the *Merodon* species of the *clavipes* species group

**Table:** 

1	Basoflagellomere orange-yellow; tibiae, tarsi and all femora in female (females of *Merodonaenigmaticus* sp. nov. and *M.rufofemoris* sp. nov. are unknown) and pro- and mesofemora in males completely or partly orange-yellowish (as in Fig. 4C, E); posterior margin of scutellum medially without long pile (Fig. 3C) (*vandergooti* subgroup)	**2**
–	Legs and basoflagellomere black to dark brown; posterior margin of scutellum with long pilosity, not interrupted medially (as in Fig. 3B) (*clavipes* subgroup)	**4**
2	Metafemur reddish yellow (Fig. 4E); anterior surstylar lobe more elongated, ~ 3.5× longer than wide (Fig. 11A: al)	***Merodonrufofemoris* sp. nov.**
–	Metafemur mostly black (as in Fig. 4C); anterior surstylar lobe shorter (as in Fig. 9A: al), < 3× longer than wide	**3**
3	Metafemur narrower and less curved, ~ 3.5× longer than wide (Fig. 4G); posterior surstylar lobe rounded (Fig. 9A: pl)	***Merodonaenigmaticus* sp. nov.**
–	Metafemur extremely broad and more curved, ~ 2.5× longer than wide (Fig. 4C); posterior surstylar lobe angular ventrally (Fig. 10A: pl)	***Merodonvandergooti* Hurkmans, 1993**
4	Wings membrane in males black, except extreme apical part (Fig. 21A); wing membrane in females with yellow veins on basal half, and with brown veins on apical half; wing along veins covered with dark brown microtrichia (Fig. 21B)	***Merodonvelox* Loew, 1869**
–	Wing mostly hyaline (as in Fig. 1)	**5**
5	Tergum 3 in male with a pair of tear drope-shape pollinose fasciate maculae separated from lateral margins (Fig. 5D); in female tergum 2 black; terga 3 and 4 with very characteristic pairs of pollinose, rounded maculae covered with dense whitish pile (Fig. 6D)	***Merodonquadrinotatus* (Sack, 1931)**
–	Tergum 3 in male with a pair of rectangular, pollinose fasciate maculae, ending close to lateral margins (as in Fig. 5A); in female tergum 2 with pair of lateral reddish yellow maculae (as in Fig. 6C) and without or with a pair of rectangular, pollinose fasciate maculae covered with grey pile on terga 3 and 4 (as in Fig. 6C)	**6**
6	Male with broad (~ 2–2.5× longer than wide) and curved metafemur (Fig. 4A); posterior surstylar lobe more straight ventrally (Fig. 8A: pl); distribution: from northern France to the Mediterranean, and from Italy through central and southern Europe to Greece, former Yugoslav countries, Albania, Romania, Ukraine, European Russia, and Turkey (Fig. 13)	***Merodonclavipes* (Fabricius, 1781)**
–	Male with less broad (~ 3–3.5× longer than wide) and less curved metafemur (Fig. 4B); posterior surstylar lobe more arcuate ventrally (Fig. 8C: pl); distribution: Iberian Peninsula and south western France (Fig. 13)	***Merodonlatens* sp. nov.**

### ﻿*Merodonpruni* species group

**Figure 23.**
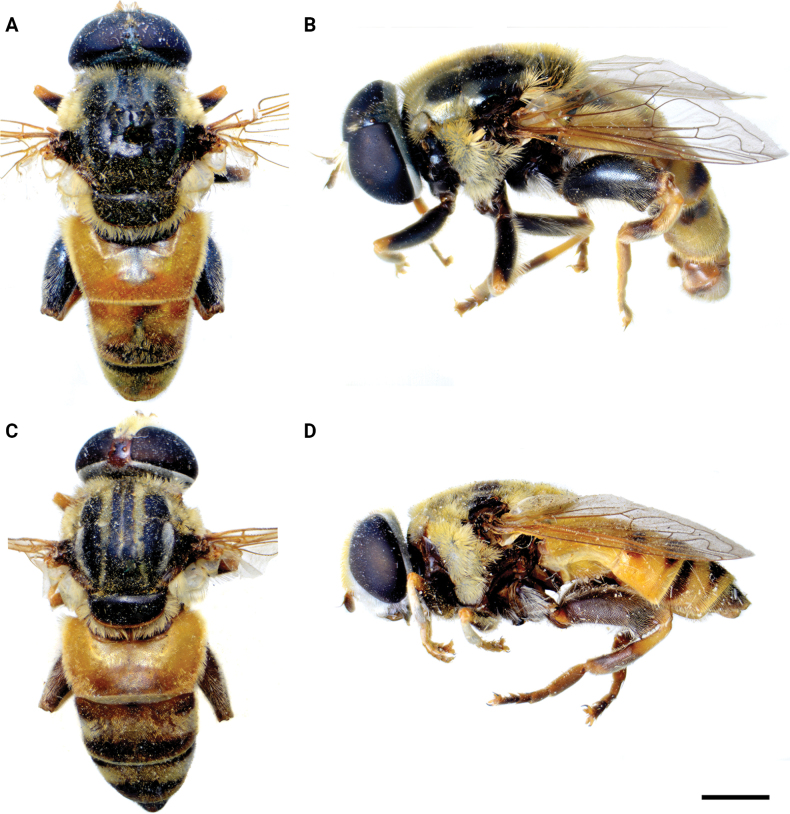
Body of *M.aequalis* sp. nov. **A, B** male **C–D** female. **A, C** dorsal view **B, D** lateral view. Scale bar: 2 mm.

**Figure 24.**
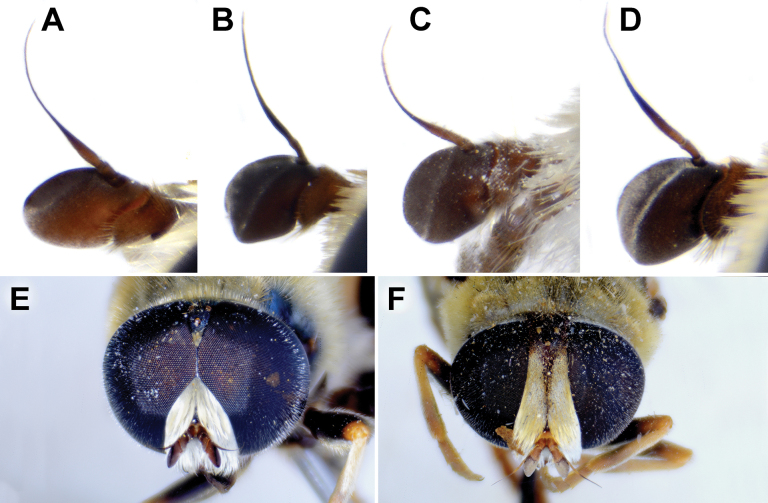
**A–D** basoflagellomera, lateral view **A***M.aequalis* sp. nov. **B***M.obscurus***C***M.pallidus***D***M.pruni*. **E, F** head od *M.aequalis* sp. nov., frontal view **A–E** male **F** female. Scale bar: 0.5 mm (**A–D**); 1 mm (**E, F**).

**Figure 25.**
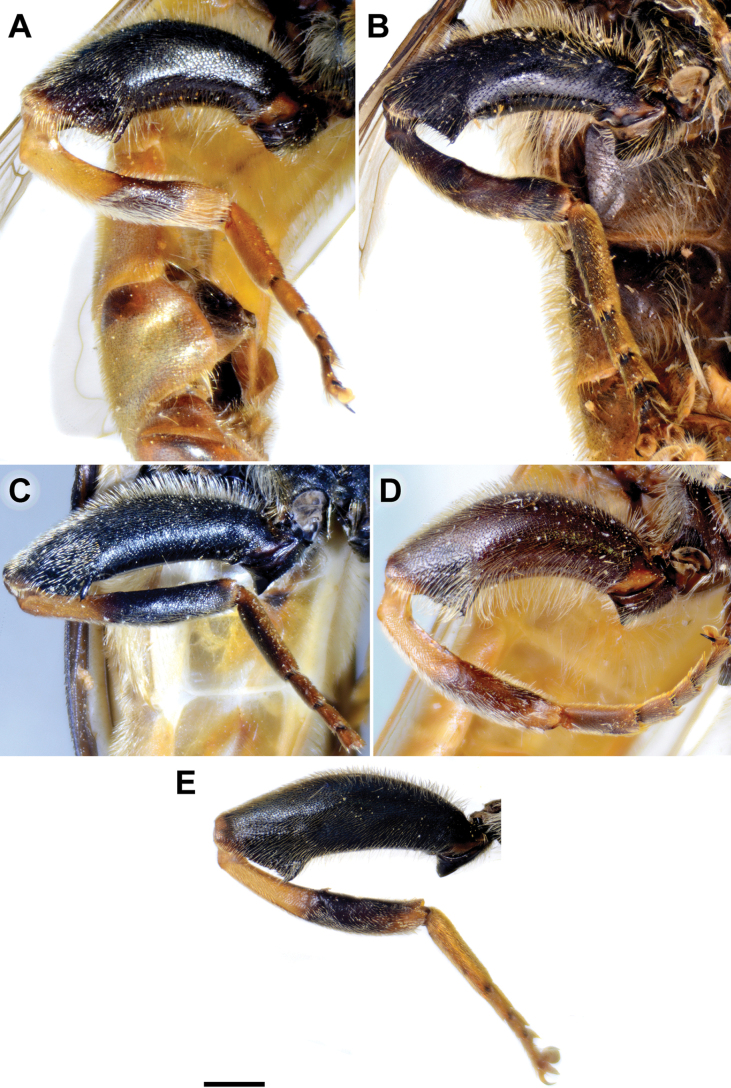
Metaleg of male, lateral view **A***M.aequalis* sp. nov. **B***M.cupreus***C***M.pruni***D***M.pallidus***E***M.obscurus*. Scale bar: 1 mm.

**Figure 26.**
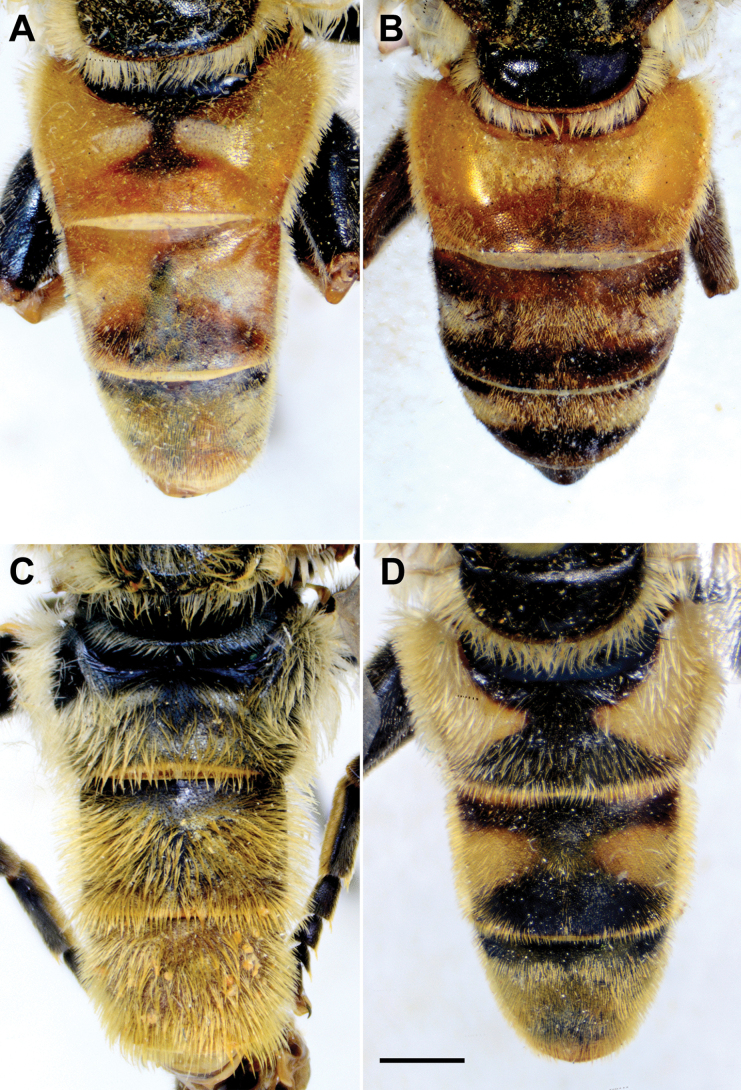
Abdomen, dorsal view **A, B***M.aequalis* sp. nov. **C***M.cupreus***D***M.pruni***A, C–D** male **B** female. Scale bar: 1 mm.

**Figure 27.**
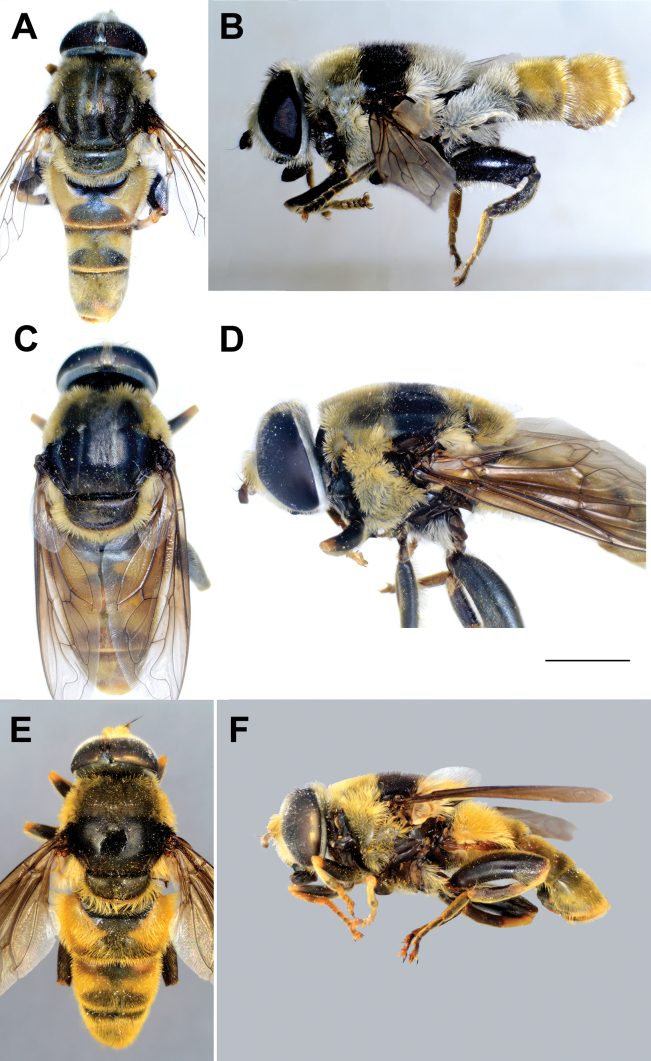
Body of male **A***M.pallidus***B***M.cupreus***C–D***M.obscurus***E, F***M.pruni***A, C, E** dorsal view **B, D, F** lateral view. Scale bar: 3 mm.

**Figure 28.**
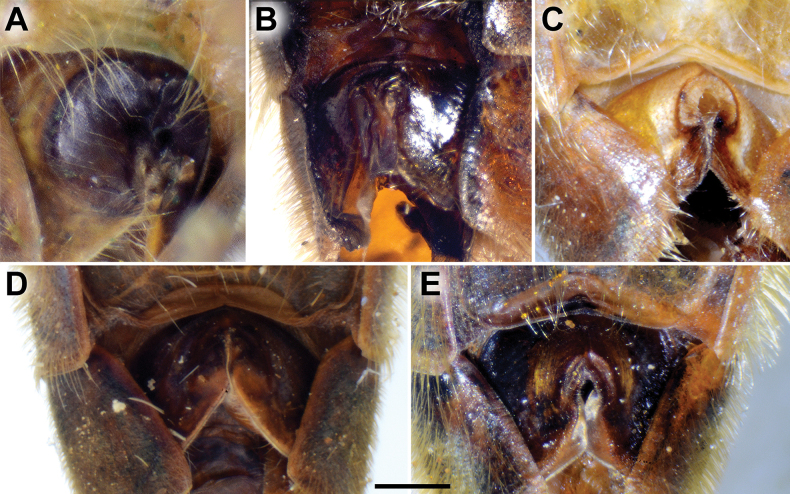
S4 of male, dorsal view **A***M.aequalis* sp. nov. **B***M.cupreus***C***M.obscurus***D***M.pallidus***E***M.pruni*. Abbreviations: al-anterior surstylar lobe, c-cercus, l-lingula, pl-posterior surstylar lobe. Scale bar: 1 mm.

**Figure 29.**
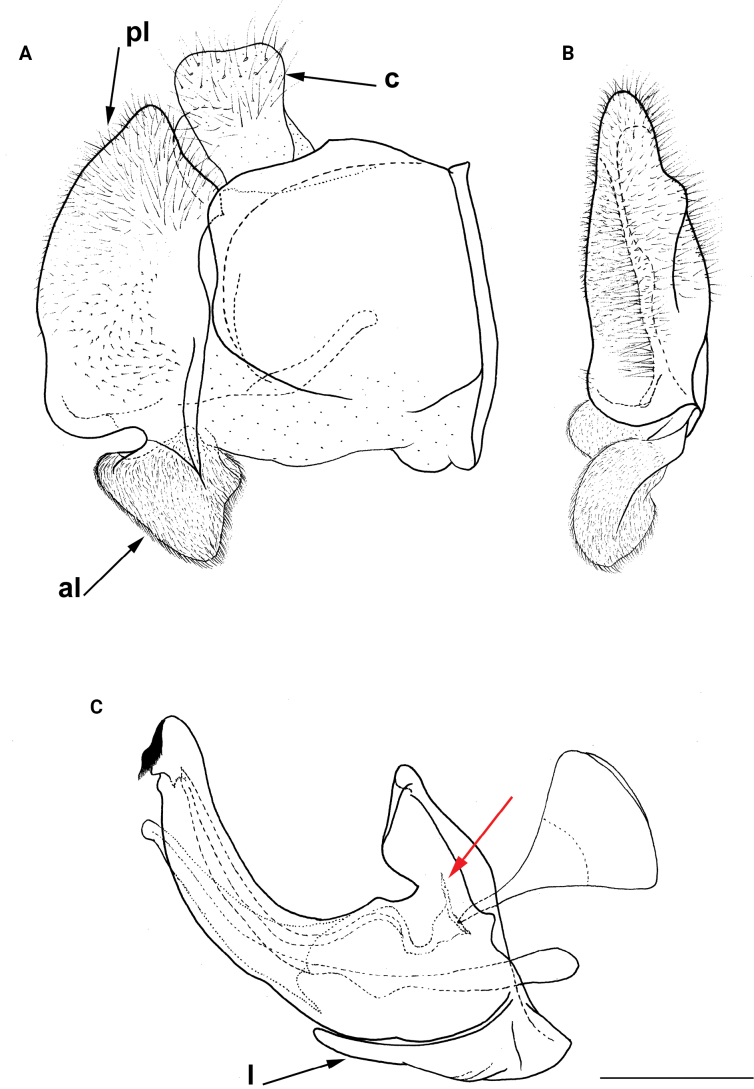
Male genitalia *M.pruni***A, B** epandrium **C** hypandrium **A, C** lateral view **B** ventral view. Filamentous prolongation on ejaculatory sack marked with red arrow. Scale bar: 0.5 mm.

**Diagnosis.** The *pruni* species group belongs to the *M.avidus*–*nigritarsis* lineage, characterised by mesocoxa without a long pile on the posterior section. This group includes large species (10–18 mm) characterised by short body pilosity (except for *M.cupreus*) especially on scutum and abdomen (as in Fig. 23), short basoflagellomere, as long as broad (Fig. 24), and pleurae usually covered with distinct whitish to yellowish pilosity; scutum with well-defined or indistinct, narrow, pollinose vittae, and some species may have a fascia with mostly black pile between wing bases; metatrochanter usually with more or less distinct calcar (Fig. 25); metafemur covered with medium to long outstanding pile (Fig. 25); tergum 2 at least partly reddish or yellow laterally (as in Fig. 26A, D), except for *M.cupreus* that has all terga black (Fig. 26C); terga 2–4 with a pair of very distinct whitish grey pollinose fasciate maculae (as in Fig. 27); sternum 4 with medial, circular incision on posterior margin (Fig. 28); male genitalia: anterior surstylar lobe small, approximately as long as wide, triangular or rectangular (as in Fig. 29A: al); posterior surstylar lobe enlarged (several times longer than wide) and broad (as in Fig. 29A: pl); cercus more or less rectangular (as in Fig. 29A: c); hypandrium with filamentous prolongation on ejaculatory sack (as in Fig. 29C: marked with red arrow); lingula medium sized and narrow (as in Fig. 29C: l). Five species belong to this species group: *Merodonpruni* is distributed in most of the Mediterranean, *M.obscurus* stat. rev. is endemic to North Africa, and the other three species are more allocated to the east, from Turkey to Israel and Pakistan.

#### ﻿ Merodon aequalis

Taxon classificationAnimaliaDipteraSyrphidae

Vujić, Radenković & Likov sp. nov.

1633D180-05BB-5E44-9967-F9DFBE95BB36

https://zoobank.org/204E1669-2E84-4938-A8B4-C30015D9B6BE

Figs 23 , 24A, E, F , 25A , 26A, B , 28A , 30A , 31A , 32 , 33 , 34

##### Type material examined.

***Holotype*.** State of Palestine • 1 ♂; Wadi Kabala Judean hills; 30 Apr. 1947; in TAU. ***Paratypes*.** Israel • 1 ♂; Golan, Qunaitra; 19 May 1983; leg. F. Kaplan; in RMNH • 1 ♂; Golan, 5 km south Qunaitra; 19.v.1983; leg. F. Kaplan; in TAU • 1 ♀; Ekron; 28 May 1921; in TAU • 1 ♀; Jerusalem; 6 May 1922; leg. P.A. Buxton; in RMNH • 1 ♂; Mrar; 14 May 1974; leg. M. Kaplan; in TAU • 1 ♀; Rehovot; 28 Sep. 1920; in RMNH • 1 ♀; Rehovot, 28 Apr. 1920; in TAU • 1 ♂, 2 ♀♀; in TAU • 1 ♂; 9 May 1925; in RMNH. State of Palestine • 1 ♂; Tikenias; 13 Oct. 1931; leg. U. Suenberg; in NHMUK • 1 ♂; 8 May; O. Theodor; in TAU.

##### Diagnosis.

**Figure 30.**
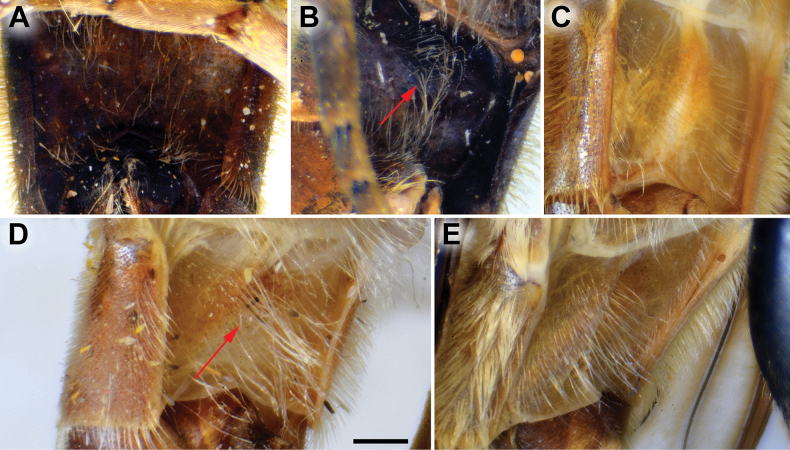
S3 of male, dorsolateral view **A***M.aequalis* sp. nov. **B***M.cupreus***C***M.obscurus***D***M.pallidus***E***M.pruni*. **B, D** area with distinct long pile marked with arrow. Scale bar: 1 mm.

**Figure 31.**
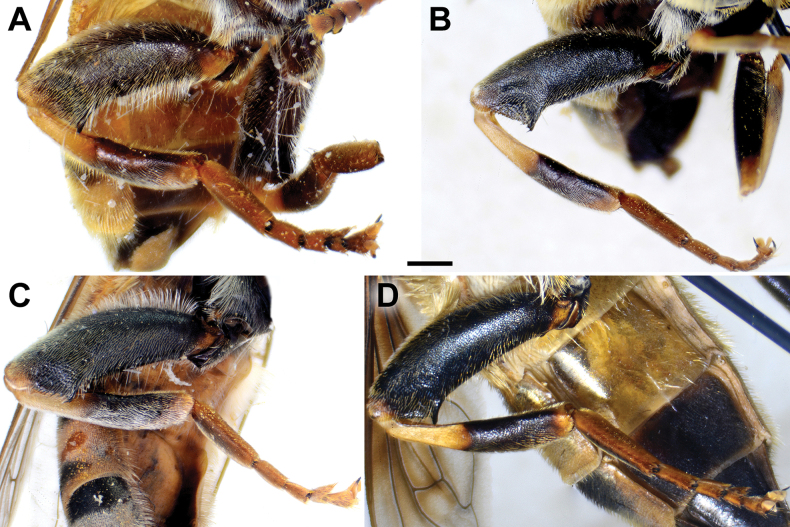
Metaleg of female, lateral view **A***M.aequalis* sp. nov. **B***M.obscurus***C***M.pallidus***D***M.pruni*. Scale bar: 1 mm.

Sternum 3 with long, equally distributed pilosity (Fig. 30A). In male the metatrochanter has a small calcar, almost absent (Fig. 25A); metafemur broad, ~ 3.5× longer than wide, strongly curved, covered with long and dense pilosity ventrally (Fig. 25A); sternum 4 on Fig. 28A. Female with rounded metatrochanter (Fig. 31A) and shorter but dense pilosity on metafemur ventrally than in male (Fig. 31A). Similar to *Merodonpallidus* stat. rev. from which differs by sternum 3 with equally distributed pilosity of the same length (Fig. 30A) (in *M.pallidus* stat. rev. with a conspicuous area of very long pilosity medially; Fig. 30D: marked with arrow), the shape of sternum 4 of male (Fig. 28A) (slightly different in *M.pallidus* stat. rev.; Fig. 28D), small calcar on metatrochanter in male, almost absent (Fig. 25A) (male of *M.pallidus* has a distinct calcar; Fig. 25D, while female of *M.pallidus* stat. rev. has the metatrochanter angular; Fig. 31C).

##### Description.

Male. Head (Fig. 24A, E). Pedicel and scapus reddish yellow; basoflagellomere from reddish yellow to brown (Fig. 24A), short, oval, ~ 1.3× longer than wide, and ~ 2× longer than pedicel, concave dorsally; fossette large, dorsolateral; arista reddish to brown and thickened at basal third; arista ~ 2.5× longer than basoflagellomere; face and frons black, with dense whitish pollinosity; face covered with dense whitish pilosity; pile on frons yellow-whitish; oral margin shiny black, without pollinosity; lunula reddish to brown, bare; eye contiguity ~ 10–12 facets long; vertical triangle isosceles, shiny, black, covered with grey-yellowish pilosity mixed with black pile around equilateral ocellar triangle; occiput with grey-yellow to whitish pile, and grey pollinose; eyes covered with short, whitish grey pile (Fig. 24E).

**Figure 32.**
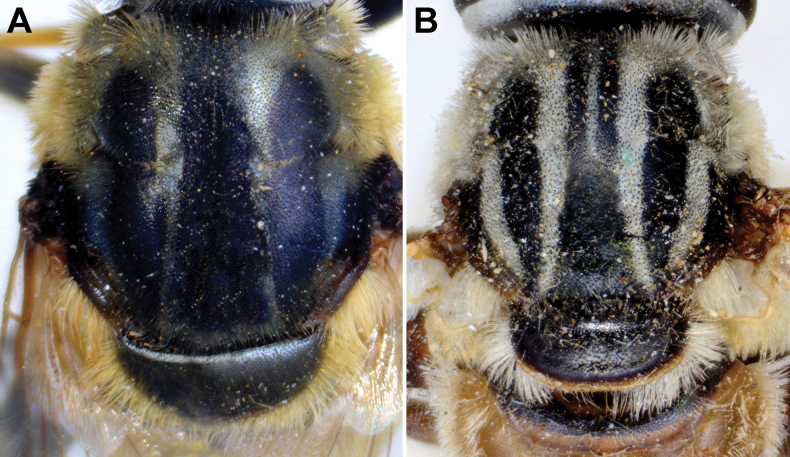
Thorax of *M.aequalis* sp. nov., dorsal view **A** male **B** female. Scale bar: 1 mm.

Thorax (Fig. 32A). Scutum and scutellum black with brownish lustre, covered with short, grey-yellow to whitish pile; pilosity between wing basis mostly black, at least around wing basis; lateral sides of scutum, excluding wing basis covered with long, golden to yellowish pile; scutum with two narrow pollinose vittae; posterior margin of scutellum with long yellowish pilosity (Fig. 32A); posterodorsal part of anterior anepisternum, posterior anepisternum (except anteroventral angle), anterior anepimeron, dorsomedial anepimeron, and posterodorsal and anteroventral parts of katepisternum with longer, dense whitish to yellow pile; wings mostly covered with microtrichia; wing veins yellowish to light brown; calypteres and halteres whitish yellow; angular calcar on metatrochanter small, almost absent; femora black except yellowish apex; metafemur broad, ~ 3.5× longer than wide, sparsely covered with long ventral pilosity (Fig. 25A); tibiae yellow to reddish, except brown medial ring; tarsi yellowish red, in some specimens brown dorsally.

Abdomen. Elongated, ~ 1.3× longer than mesonotum; tergum 1 black, terga 2–4 reddish yellow, medially partly brown; terga with a pair of broad, distinct silver-grey pollinose fasciate maculae; pile on terga yellow to whitish, medially short, adpressed, in some specimens black pile present on dark parts of terga 3 and 4 medially (Fig. 26A); sterna brown, covered with long, equally distributed whitish pile (Fig. 30A); posterior margin of sternum 4 with characteristic posteromedial circular incision (Fig. 28A).

**Figure 33.**
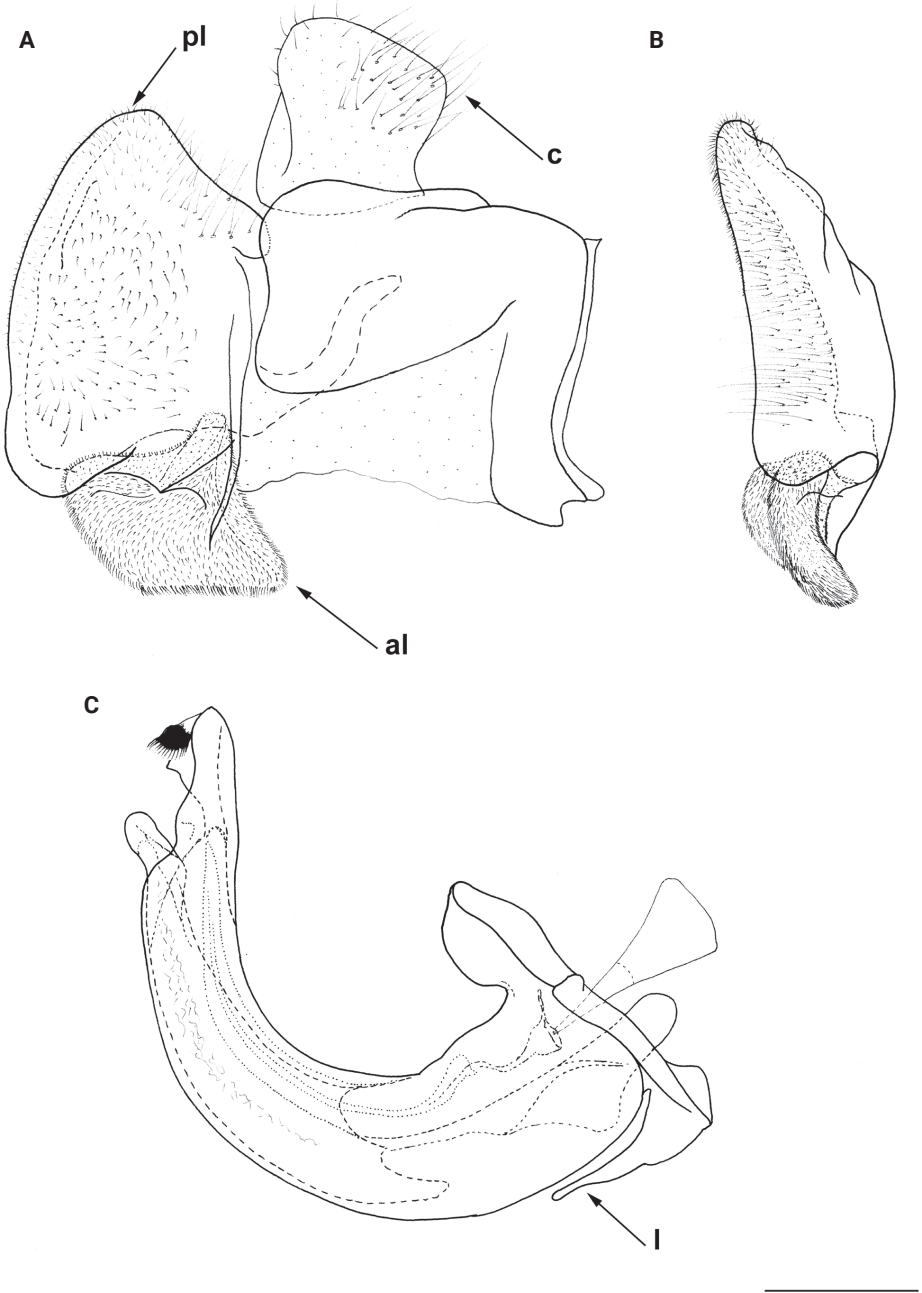
Male genitalia *M.aequalis* sp. nov. **A, B** epandrium **C** hypandrium. **A, C** lateral view **B** ventral view. Abbreviations: al-anterior surstylar lobe, c-cercus, l-lingula, pl-posterior surstylar lobe. Scale bar: 0.5 mm.

Male genitalia (Fig. 33). Anterior surstylar lobe rectangular (Fig. 33A: al); posterior surstylar lobe large and broad, ~ 1.5× longer than wide (Fig. 33A: pl); cercus rectangular (Fig. 33A: c); hypandrium sickle-shaped, without lateral projections; lingula short (Fig. 33C: l).

Female. Similar to the male except for normal sexual dimorphism and the following characteristics: frons with broad pollinose vittae along eyes or completely pollinose, and reddish at the level of the ocellar triangle (Fig. 24F); scutum with five distinct pollinose vittae (Fig. 32B); metatrochanter rounded; pilosity on the ventral surface of metafemur shorter but denser than in male (Fig. 31A); tergum 2 all reddish, while terga 3–5 more brownish (Fig. 26B).

##### Distribution and biology.

**Figure 34.**
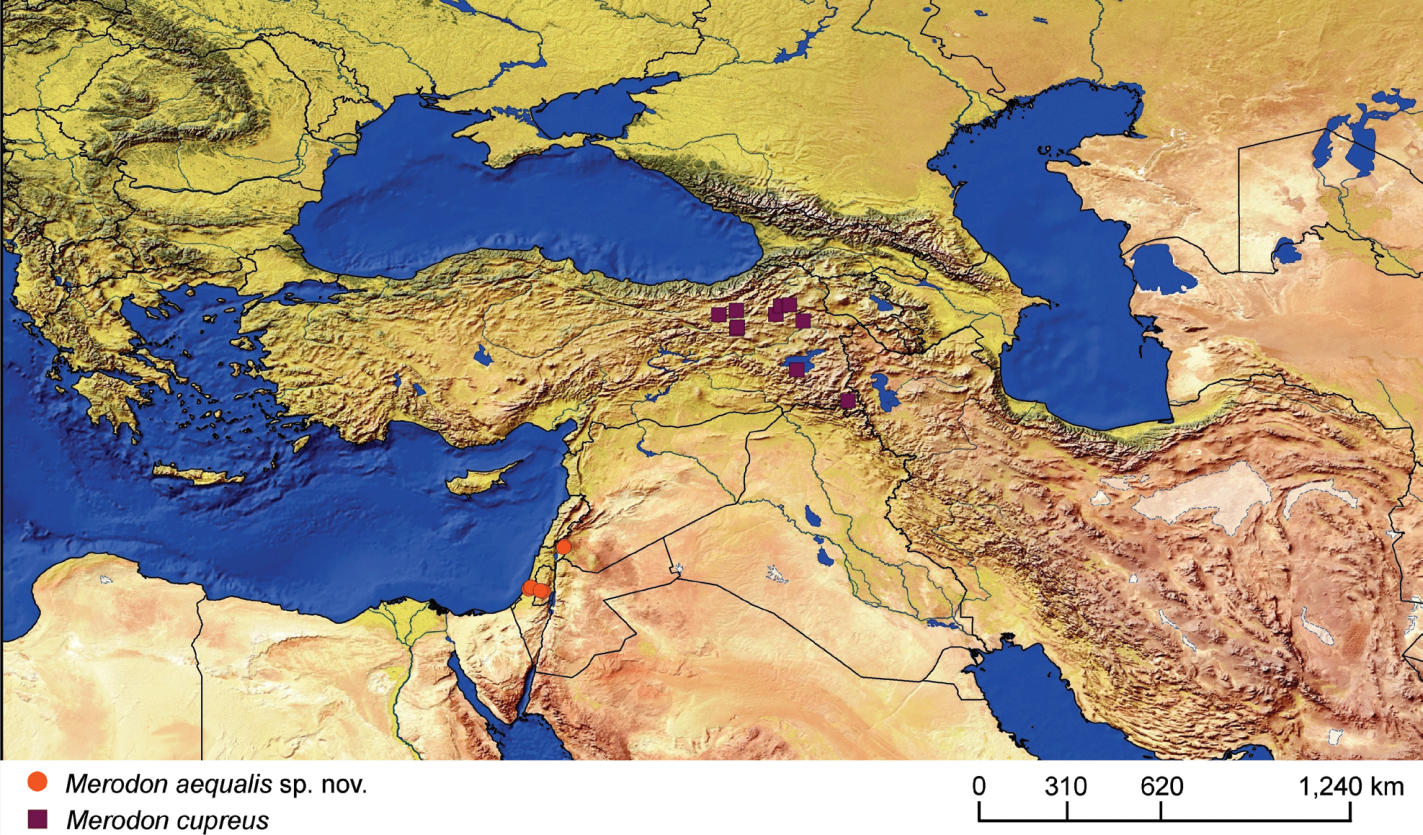
Distribution map of *Merodonaequalis* sp. nov. and *M.cupreus*.

The range is restricted to Israel and the State of Palestine (Fig. 34). Its preferred environment is Eastern Mediterranean conifer-sclerophyllous-broadleaf forests. The vegetation of this ecoregion includes maquis, coniferous forests of *Pinushalepensis* Mill. and *P.brutia* Ten., dry *Quercus* spp. woodlands and steppe formations (WWF 2022). Flight period: April/October. Developmental stages: not described.

**Figure 35.**
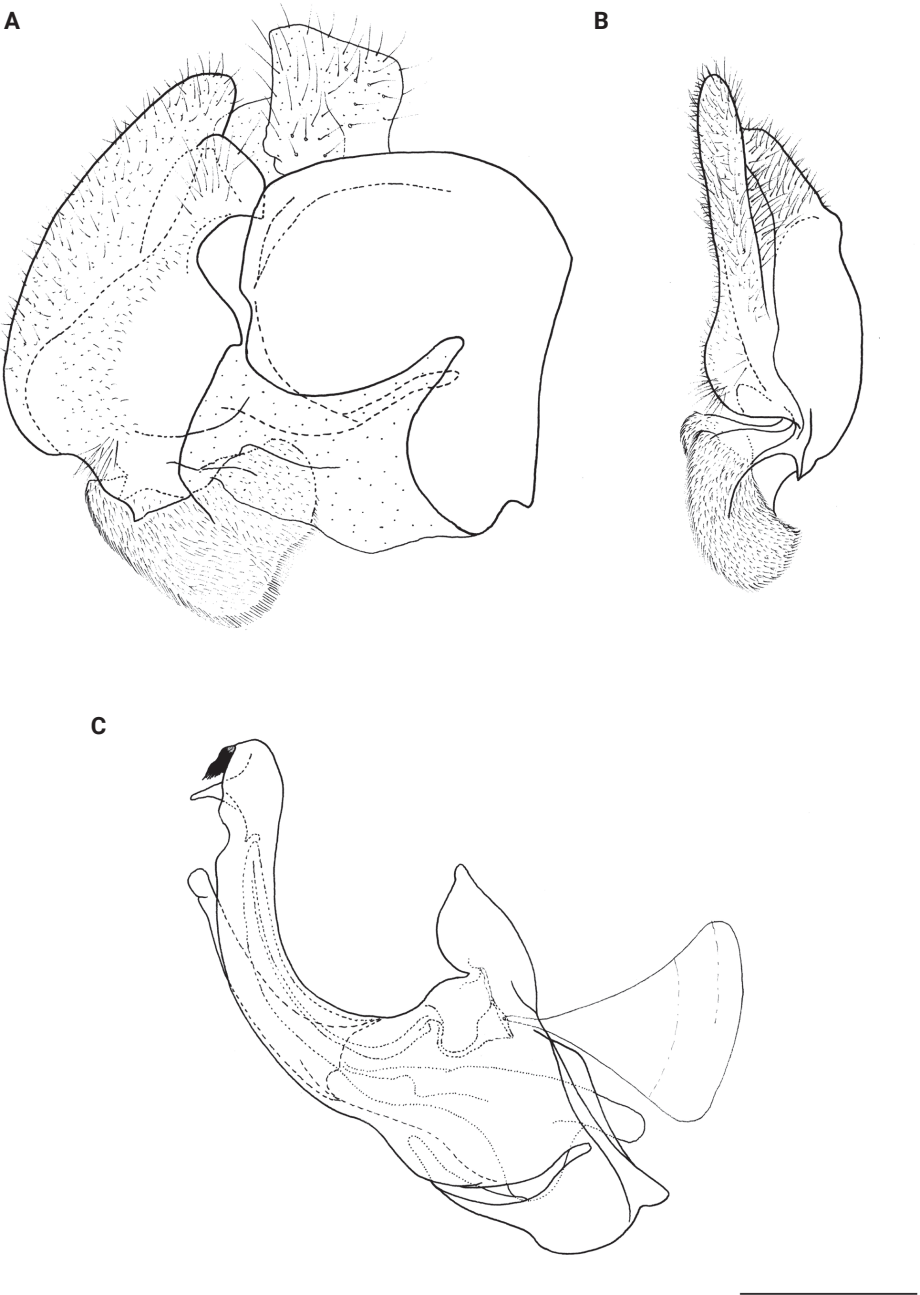
Male genitalia *M.cupreus***A, B** epandrium **C** hypandrium **A, C** lateral view **B** ventral view. Scale bar: 0.5 mm.

##### Etymology.

Adjective *aequalis* meaning equal, similar, refers to the equally distributed pilosity of the same length on sternum 3 in males opposite to the related species *Merodonpallidus* stat. rev. with a conspicuous area of very long pilosity medially. Species epithet to be treated as an adjective.

#### ﻿ Merodon cupreus

Taxon classificationAnimaliaDipteraSyrphidae

Hurkmans, 1993

EA5FF18B-8936-52B8-8AC7-B7928DE3CFBE

Merodon cupreus Hurkmans, 1993: 179.

##### Type locality.

Turkey, “Kars”. Original description was based on a male holotype and a high number of male and female paratypes (all in RMNH) (Hurkmans 1993: 179). Holotype (designated by Hurkmans): male, Turkey, Kars (RMNH), [specimen dry pinned]. Original labels: [Turkey, Kars, Handere 2100–2200 m, 20 km W of Saricamiş, 1.viii.1983, leg. J. A. W. Lucas], [Holotype of *Merodoncupreus* Hurkmans] (examined).

##### Diagnosis.

Bumble bee mimic species (similar to species from *clavipes* species group) with pile on scutum longer than basoflagellomere (shorter in other species of the *pruni* species group); mesonotum with whitish pile except for broad black-pilose fascia between wing bases (Fig. 27B); tergum 2 black (mostly reddish yellow in other species of the *pruni* group); tergum 2 with whitish to yellow pile, and terga 3 and 4 covered with yellow to reddish pilosity (Fig. 26C); legs black; calcar on metatrochanter distinct; metafemur curved and covered with long, dense pilosity (Fig. 25B); sternum 3 medially with distinct pilosity (Fig. 30B: marked with arrow); sternum 4 in Fig. 28B. Male genitalia in Fig. 35. Similar to *Merodonclavipes* and *M.quadrinotatus* from which it clearly differs by its short basoflagellomere, which is as long as broad (as on Fig. 24A) (basoflagellomere > 2× longer than wide in *M.clavipes* (Fig. 2A) and *M.quadrinotatus* (Fig. 2C)).

##### Distribution and biology.

The species is solely distributed in Turkey (Fig. 34; Suppl. material 2), including the eastern Pontic and Taurus mountains belonging to the Irano-Anatolian hotspot. These chains of high mountains form a natural barrier between the Mediterranean Basin and the dry plateaux of Western Asia. This topographically complex and extensive system of mountains and closed basins includes major parts of central and eastern Turkey. Historically, the mountains have served both as refuge and corridor between the eastern Mediterranean and western Asia, giving rise to multiple patches of local endemism. The principal habitat of the species inside the hotspot is mountainous forest steppe, supporting oak-dominant (*Quercus* spp.) deciduous forests (CEPF 2022). Flight period: June/August. Developmental stages: not described.

#### ﻿ Merodon obscurus

Taxon classificationAnimaliaDipteraSyrphidae

Gil Collado, 1929 stat. rev.

3B3B3089-93D1-5DCD-98DA-2D2569ACDB25

Merodon pruni var. obscurus Gil Collado, 1929: 407.

##### Type locality.

Morocco (“Tanger”). *Merodonobscurus* was described as a variety of *M.pruni*. Holotype: male, Morocco, (MNCN) [specimen dry pinned]. Original label: [Tanger, Mz. Escalera / M.prunivar.obscurus Gil Tipo, Gil Collado det. / M.N.C.N. Madrid] (examined).

##### Notes.

This species was listed as synonym of *Merodonpruni* by Peck (1988: 173) and Hurkmans (1993: 185). Based on our morphometry and molecular data, this is a valid taxon distributed in North West Africa, far from the range of *M.pruni* in the Eastern Mediterranean (Fig. 37).

##### Diagnosis.

**Figure 36.**
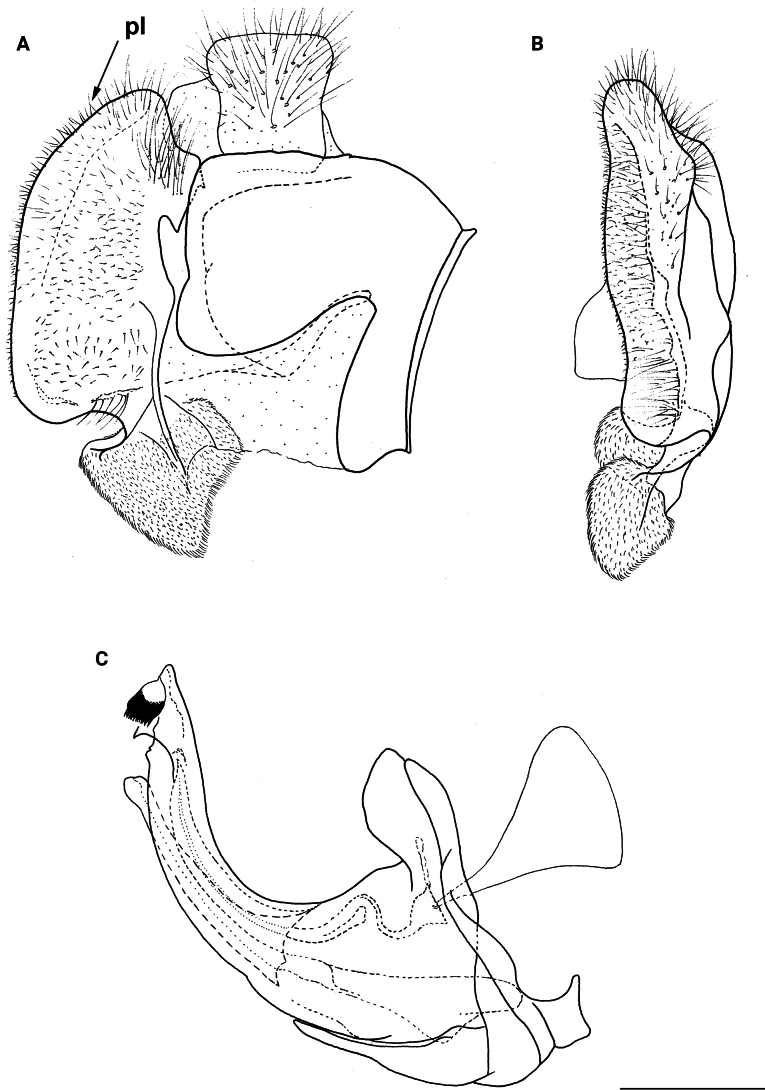
Male genitalia *M.obscurus***A, B** epandrium **C** hypandrium **A, C** lateral view **B** ventral view. Abbreviations: pl-posterior surstylar lobe. Scale bar: 0.5 mm.

**Figure 37.**
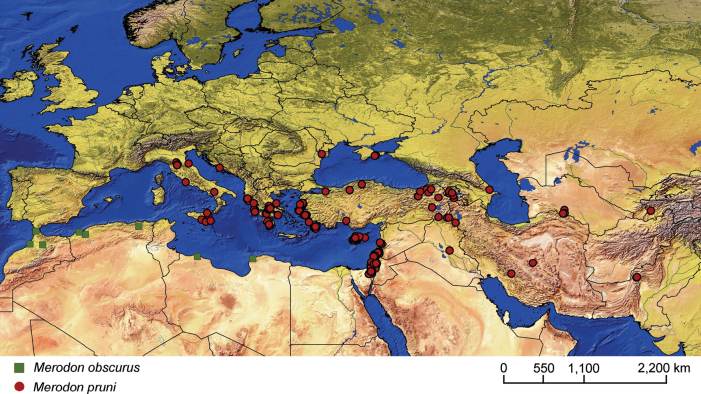
Distribution map of *Merodonpruni* and *M.obscurus*.

Sternum 3 with long, equally distributed pilosity (Fig. 30C). In male calcar at metatrochanter distinct (Fig. 25E); metafemur medium broad, ~ 5× longer than wide, with ventral margin slightly curved and covered with sparse pilosity ventrally (Fig. 25E); sternum 4 in Fig. 28C. Female with angular metatrochanter and sparse pile on metafemur ventrally (Fig. 31B). Male genitalia in Fig. 36. Similar to *Merodonpruni* except for the posterior surstylar lobe that is broader (~ 2.2× longer than wide) and more rounded apically (Fig. 36A: pl) (in *M.pruni* the posterior surstylar lobe is ~ 2.5× longer than wide and tapering to the tip; Fig. 29A: pl). *Merodonobscurus* stat. rev. occurs in North Africa, while *M.pruni* is an Eastern Mediterranean species (Fig. 37). Molecular and morphometric data clearly separated these two species (Figs 15, 16, 17, Suppl. material 3).

##### Distribution and biology.

This species occurs in Algeria, Libya and Morocco (Fig. 37; Suppl. material 2). The preferred environment of *Merodonobscurus* stat. rev. includes sparsely-vegetated open ground and dry/semi-arid grassland with scattered tall herbs. Flowers visited: *Ferula*, *Foeniculum*. Flight period: April/September. Developmental stages: not described.

#### ﻿ Merodon pallidus

Taxon classificationAnimaliaDipteraSyrphidae

Macquart, 1842 stat. rev.

E4B2B9DF-9C76-5C3E-94B5-5F60A43D3F0C

Merodon pallidus Macquart, 1842: 70.

##### Type locality.

Iraq (Baghdad). The original description was based on a single female specimen (holotype identified by Vockeroth in 1969, unpublished). The holotype is located in the Paris Museum (MNHN): female, Iraq, Baghdad, [specimen dry pinned]. Original labels: [No. 1187. / *Merodon* / *pallidus*] [label handwritten], [Bagdad] [label handwritten], [HOLOTYPE / Vockeroth ‘69’, ‘*Merodonpallidus* / Macquart 1842 / det. Vujić 2008] [red label] (examined).

##### Notes.

Peck (1988: 173) and Hurkmans (1993: 185) cited *Merodonpallidus* as a synonym of *M.pruni*. Hurkmans (1993: 185) designated a “lectotype” of *M.pallidus* based on incorrect interpretation of a male specimen from Baghdad deposited in an unknown collection. *Merodonpallidus* was described based on one female and there are no indications that the specimen mentioned in Hurkmans (1993) belongs to the type material. A lectotype may be designated from syntypes (ICZN 1999), but Hurkmans “lectotype” was erroneously designated as the type. The identity of the Hurkmans “lectotype” could not be validated because this specimen is not located in any museum. Based on our assessment of morphological data, *M.pallidus* is a valid taxon, which we redefine herein. Based on our analysis of material belonging to distinct individuals collected from Iran, Israel, Pakistan, Palestine and Turkey (10 females, 7 males), the females are conspecific with the holotype of *M.pallidus*, so we re-describe the male herein.

##### Diagnosis.

**Figure 38.**
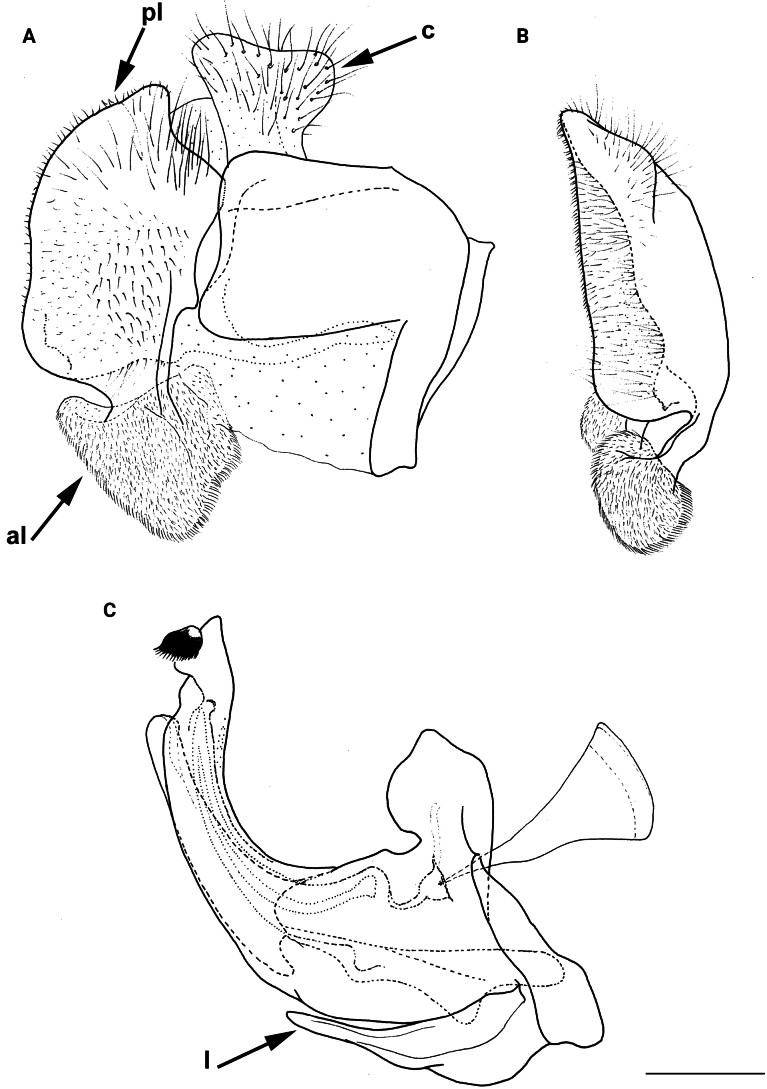
Male genitalia *M.pallidus***A, B** epandrium **C** hypandrium **A, C** lateral view **B** ventral view. Abbreviations: al-anterior surstylar lobe, c-cercus, l-lingula, pl-posterior surstylar lobe. Scale bar: 0.5 mm.

Sternum 3 with long and dense pile medially (Fig. 30D: marked with arrow). In male the metatrochanter has a less distinct calcar (Fig. 25D); metafemur broad (~ 3× longer than wide), strongly curved, covered with long and dense pilosity ventrally (Fig. 25D); sternum 4 in Fig. 28D. Female with angular metatrochanter and long and sparse pile on metafemur ventrally (Fig. 31C). Male genitalia in Fig. 38. Similar to *Merodonaequalis* sp. nov. from which differs by sternum 3 with an area of long pilosity medially (Fig. 30D: marked with arrow) (in *M.aequalis* sp. nov. sternum 3 has equally distributed pilosity of the same length; Fig. 30A); the shape of sternum 4 of male (Fig. 28D), which is slightly different in *M.aequalis* sp. nov. (Fig. 28A); and a distinct calcar on the metatrochanter of the male (Fig. 25D) and female with an angular metatrochanter (Fig. 31C) (in *M.aequalis* sp. nov. the calcar is almost absent in both sexes; Figs 25A, 31A).

##### Re-description.

Male. Head. Pedicel and scapus reddish yellow; basoflagellomere from reddish yellow to brown (Fig. 24C), short, oval, ~ 1.3× longer than wide, and ~ 2× longer than pedicel, concave dorsally; fossette large, dorsolateral; arista reddish to brown and thickened at basal third; arista ~ 2.5× longer than basoflagellomere; face and frons black, with dense whitish pollinosity; face covered with dense whitish pilosity; pile on frons yellow-whitish; oral margin shiny black, with sparse pollinosity; lunula reddish to brown, bare; eye contiguity ~ 12 facets long; vertical triangle isosceles, shiny, black, covered with grey-yellowish pilosity; ocellar triangle isosceles; occiput with grey-yellow to whitish pile, and grey pollinose; eyes covered with short, whitish grey pile.

Thorax. Scutum and scutellum black with brownish lustre, covered with short, greyish white pile; pilosity near wing bases mostly black; lateral sides of scutum covered with long, golden to the greyish white pile; scutum with five distinct pollinose vittae (Fig. 27A); posterior margin of scutellum with long pilosity; posterodorsal part of anterior anepisternum, posterior anepisternum (except anteroventral angle), anterior anepimeron, dorsomedial anepimeron, and posterodorsal and anteroventral parts of katepisternum with dense greyish white pile; wings mostly covered with microtrichia; wing veins yellowish to light brown; calypteres and halteres whitish yellow; angular calcar on metatrochanter distinct; femora black except yellowish apex; metafemur broad, ~ 3× longer than wide, covered with long whitish pilosity (Fig. 25D); tibiae yellow to reddish, except brown medial ring; tarsi yellowish red, in some specimens brown dorsally.

Abdomen. Elongated, ~ 1.3× longer than mesonotum; tergum 1 black, terga 2–4 usually reddish yellow, in some specimens medially partly black; terga with a pair of broad, distinct silver-grey pollinose fasciate maculae (Fig. 27A); pile on terga whitish, medially short, adpressed; sterna brown, covered with long, whitish pile; sternum 3 with an area of long pilosity medially (Fig. 30D: marked with arrow); posterior margin of sternum 4 with characteristic medial circular structure (Fig. 28D).

Male genitalia (Fig. 38). Anterior surstylar lobe triangular (Fig. 38A: al); posterior surstylar lobe large and broad (~ 2× longer than wide) (Fig. 38A: pl); cercus trapezoid (Fig. 38A: c); hypandrium sickle-shaped, without lateral projections; lingula long (Fig. 38C: l).

Female. Similar to the male except for normal sexual dimorphism and the following characteristics: frons covered with whitish pollinosity; scutum between wing bases with more black pilosity; metafemur narrower (~ 3.5× longer than wide), with ventral pilosity shorter than in male (Fig. 31C); terga 3 and 4 with short adpressed black pilosity medially on dark parts.

##### Distribution and biology.

**Figure 39.**
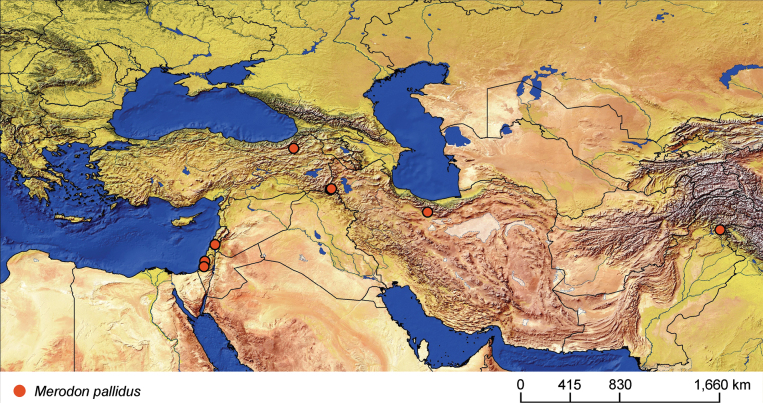
Distribution map of *Merodonpallidus*.

The species range includes Iran, Israel, Pakistan, the State of Palestine and Turkey (Fig. 39; Suppl. material 2). In Iran, it has been recorded within arid and semi-arid forest ecosystems where *Quercusbrantii* is the dominant vegetation type (Azizi Jalilian et al. 2020) belonging to the Elburz range forest steppe ecoregion (Olson et al. 2001). The western part of the range of *Merodonpallidus* (Turkey, State of Palestine and Israel) belongs to the Eastern Mediterranean conifer-sclerophyllous-broadleaf forests ecoregions The vegetation of this ecoregion includes maquis, coniferous forests of *Pinushalepensis* Mill. and *P.brutia* Ten., dry *Quercus* spp. woodlands and steppe formations (WWF 2022). In Pakistan, *M.pallidus* occurs in warm conifer/mixed forests (Siddiqui et al. 1999). Flight period: April/August. Developmental stages: not described.

***Merodonpruni* (Rossi, 1790)**

*Syrphuspruni* Rossi, 1790: 293.

*Merodonfulvus* Macquart, 1834: 514.

*Merodonsicanus* Rondani, 1845: 258, 264.

*Merodonfuscinervis* Von Röder, 1887: 73.

***Syrphuspruni* Rossi, 1790: 293**

**Type locality.** Italy (Toscana). The original description was based on an unspecified number of syntypes (Rossi 1790: 293). Type material could not be traced ‘in provinciis Florentina et Pisana’ [Firenze and Pizza, Italy] [not located, not examined]. Based on the description and figure from the original publication (Rossi 1790), the identity of types is clear and fits the actual concept of species presented in Hurkmans (1993: 185). This species was cited in recent European publications (e. g. Speight 2020; Vujić et al. 2021a).

***Merodonfulvus* Macquart, 1834: 514**

**Type locality.** France (“France méridionale”). Synonymy with *Merodonpruni* was cited in Sack (1931), Peck (1988: 172) and Hurkmans (1993: 185). Type material presumably lost.

***Merodonsicanus* Rondani, 1845: 258, 264**

**Type locality.** Italy, “Sicilia”. The original description was based on two female syntypes. One syntype was designated as a lectotype by Hurkmans (1993: 185): Original label [58] [number referring to the description of *Merodonsicanus* in the museum’s catalogue of Rondani collection]. This designation was based on syntype (examined) deposited in the LSF.

***Merodonfuscinervis* Von Röder, 1887: 73**

**Type locality.** Greece (“Crete”). Synonymy with *Merodonpruni* was cited in Sack (1913), Peck (1988) and Hurkmans (1993). Type material presumably lost.

**Diagnosis.** Sternum 3 with more or less equally distributed pilosity (Fig. 30E). In male calcar at metatrochanter distinct (Fig. 25C); metafemur medium broad (~ 4.5× longer than wide), ventral margin slightly curved, and covered with sparse pilosity ventrally (Fig. 25C); sternum 4 in Fig. 28E. Female with angular metatrochanter and sparse pile on metafemur ventrally (Fig. 31D). Male genitalia in Fig. 29. Similar to *Merodonobscurus* stat. rev. from which differs by posterior surstylar lobe tapering to the tip (Fig. 29A: pl) (rounded apically in *M.obscurus* stat. rev.; Fig. 36A: pl) and its distribution in the Eastern Mediterranean (*M.obscurus* stat. rev. is restricted to North Africa).

**Distribution and biology.** It occurs throughout much of southern Europe (Italy, Croatia, Greece, Cyprus, Romania), eastwards to Ukraine, Turkey, Armenia, Azerbaijan, Iran, Iraq, Israel, State of Palestine, Lebanon, Pakistan, Turkmenistan, and Tajikistan. Hurkmans (1993) lists North Africa as part of the species range, but those specimens most likely belong to *Merodonobscurus*. Speight (2020) also mentions Austria and southern France (with the remark that it is most probably extinct), but species presence in those countries could not be confirmed (Fig. 37; Suppl. material 2). The preferred environment of species *M.pruni* is sparsely-vegetated open ground, dry/semi-arid grassland with scattered tall herbs, open areas in low-altitude *Abiescephalonica* forest on limestone, and *Castanea* forest (Speight 2020). At the northern edge of its range, i.e., in Ukraine, the species occurs in steppe habitats. Hurkmans (1985) provides some information on male territorial behaviour; also stating that females fly fast and very close to the ground and are much less noticeable than the males. Both sexes fly silently (Speight 2020). Flowers visited: *Ferula*, *Foeniculum*. Flight period: May/October, with peaks in May and September. Developmental stages: not described (Speight 2020).

### ﻿Key for the *Merodon* species of the *pruni* species group

(The separation of females of *Merodonpruni* and *M.obscurus* is uncertain based on morphological characters, but it can be done based on molecular and morphometric data and by the geographic range)

**Table:** 

1	Bumble bee mimic species (Fig. 27B) with pile on scutum longer than basoflagellomere; tergum 2 black (Fig. 26C); mesonotum with whitish pile, except broad black-pilose fascia between wing bases (Fig. 27B); tergum 2 with whitish pile; terga 3 and 4 covered with yellow to reddish pilosity (Fig. 26C); legs black; calcar on metatrochanter distinct (Fig. 25B); metafemur curved, covered with long, dense pilosity (Fig. 25B); sternum 3 medially with distinct pilosity; sternum 4 as in Fig. 28B	***Merodoncupreus* Hurkmans, 1993**
–	Species with shorter body pilosity; pile on scutum shorter than basoflagellomere; tergum 2 mostly reddish	**2**
2	Metafemur with sparse ventral pilosity (as in Fig. 25C)	**4**
–	Metafemur with long and dense ventral pilosity (as in Fig. 25D)	**3**
3	Sternum 3 medially with equally distributed pilosity (Fig. 30A); sternum 4 of male in Fig. 28A; calcar on metatrochanter in male small, almost absent (Fig. 25A); in female metatrochanter rounded (Fig. 31A)	***Merodonaequalis* sp. nov.**
–	Sternum 3 with area of long pilosity medially (Fig. 30D: marked with arrow); sternum 4 of male in Fig. 28D; calcar on metatrochanter in male distinct (Fig. 25D); in female metatrochanter angular (Fig. 31C)	***Merodonpallidus* Macquart, 1842, stat. rev.**
4	Posterior surstylar lobe tapering to the tip (Fig. 29A: pl); distribution: Eastern Mediterranean	***Merodonpruni* (Rossi, 1790)**
–	Posterior surstylar lobe more rounded apically (Fig. 36A: pl): distribution: North Africa	***Merodonobscurus* Gil Collado, 1929, stat. rev.**

### ﻿Molecular analyses

The molecular analyses of the two studied *Merodon* species groups involved 72 nucleotide sequences in total including outgroups. We studied the dataset of the concatenated 3′ and 5′ fragments of the COI gene which comprised a total of 1273 characters (612 nucleotide positions of 5′-end fragment of COI gene and 661 of 3′-end fragment of this gene), of which 336 were parsimony informative. All positions containing missing data were excluded from the analysis. In the analyses, we involved the representatives of previously described *Merodon* lineages by Vujić et al. (2021a). All five lineages clearly resolved as clades on both obtained trees, Maximum Parsimony (Fig. 15) and Maximum Likelihood (Suppl. material 3): *natans* (with bootstrap value MP = 98, ML = 99), *albifrons* (MP = 91, ML = 98), *desuturinus* (MP = 78, ML = 83), *aureus* (MP = 99, ML = 98), and *avidus–nigritarsis* lineage (MP = 79, ML = 96). Within M. *avidus–nigritarsis* lineage, both analysed species groups resolved as monophyletic with high bootstrap support (MP = 100 and ML = 99 for both groups). Comparing MP and ML trees, applied methods resulted in similar tree topologies within analysed species groups, with slight differences in bootstrap values. Within *clavipes* group samples from Spain clearly separated (with bootstrap support MP = 80 and ML = 94) from the other analysed species of the group (*M.clavipes* and *M.velox*). This confirms the existence of additional new species of the group, named *M.latens* sp. nov. *Merodonobscurus* is proved to be valid species and is resolved in a separated clade within the *pruni* species group with 99 bootstrap support value on the two inferred trees, and clearly distinct from *M.pruni*.

### ﻿Geometric morphometrics

Our species-based discriminant analysis (DA) provided evidence for highly significant wing shape differences among all species pairs (Table 1). Additionally, cross-validation of that analysis based on wing shape revealed highly accurate species assignment (95.4%). Of 87 specimens, only four were misclassified: one *Merodonobscurus* as *M.pruni*, two *M.latens* sp. nov. as *M.clavipes* and one *M.clavipes* as *M.latens* sp. nov. All specimens of *pruni* species group were correctly classified. We obtained a congruent classification based on the Gaussian naive Bayes classifier, with two *M.obscurus* misclassified as *M.pruni*, one *M.latens* sp. nov. as *M.clavipes* and two *M.clavipes* as *M.latens* sp. nov.

The species-based CVA conducted on wing shape parameters generated three highly significant canonical axes (CV1: Wilks’ = 0.01199, *χ2* = 331.7617, *p* < .01; CV2: Wilks’ = 0.27443, *χ2* = 96.9786, *p* < .01; CV3: Wilks’ = 0.54099, *χ2* = 46.0769, *p* < .01). The first canonical axis represents the majority of wing shape variation (92%) and clearly differentiates the *clavipes* and *pruni* groups (Fig. 16A, B). The second and third axes reflect intra-group variability and they clearly differentiated species *M.latens* sp. nov. from *M.clavipes* and *M.obscurus* from *M.pruni* (Fig. 16A, B). The same pattern of wing shape similarity was depicted in the phenogram based on squared Mahalanobis distances (Fig. 16C).

Pairwise differences in average wing shape were visualised for species within the groups using superimposed outline drawings (Fig. 16D). Differences inside the *clavipes* group were attributable to displacements of all landmarks. In contrast, differences between species *M.pruni* and *M.obscurus* were associated with highly prominent landmark displacements in central and distal parts of their wings (Fig. 16D).

### ﻿Population-level geometric morphometrics analysis

Our population-based DA generated an overall correct classification of 89.8% for the specimens. All *Merodonobscurus* were correctly classified, whereas all misclassified specimens of *M.pruni* (4 out of 40) were assigned to conspecific populations. Regarding *M.latens* sp. nov., only two specimens out of ten were misclassified as *M.clavipes* from Rhodope, Greece. All specimens of *M.clavipes* were correctly classified.

Our population-based CVA produced four significant CV axes, from which the first three were informative in species delimitation. The first CV axis describes differences in wing shape between the *clavipes* and *pruni* species groups (Fig. 17A, B). Moreover, CV1 indicated wing shape differences between species *M.latens* sp. nov. and *M.clavipes* from Rhodope, Greece (Fig. 17A). CV2, representing 8% of total shape variation, clearly separated *M.pruni* populations from *M.obscurus* (Fig. 17A). This axis also clearly separated *M.clavipes* specimens from Crete, Greece, from both *M.clavipes* specimens from Rhodope, Greece and *M.latens* sp. nov. (Fig. 17A). The third axis, representing 3% of total shape variation, separated species in the *clavipes* group (Fig. 17B).

We used a UPGMA phenogram constructed from squared Mahalanobis distances to summarise differences in wing shape among the investigated populations (Fig. 17C). The resulting phenogram revealed two main clusters, one for the *clavipes* group and another for the *pruni* group (Fig. 17C). All conspecific populations were grouped within their respective cluster.

## ﻿Discussion

### ﻿Systematics and taxonomy

The *clavipes* and *pruni* species groups comprise large hoverfly species, indeed the largest in size of the *avidus*–*nigritarsis* lineage. Bumble bee-like taxa from the *clavipes* group are characterised by their long body pilosity and elongated basoflagellomere (> 2× longer than wide), contrasting with the short body pilosity and short basoflagellomere (approximately as long as wide) of species in the *pruni* group. The nominal species of this latter group, *M.pruni*, is covered with very short pile, although one group representative (*M.cupreus*) exhibits an extremely similar appearance to the bumble bee-like species of the *clavipes* group. Representatives of both groups possess varying structures of the basoflagellomere and male genitalia, especially in terms of the shape of the surstylar lobe, which is characteristic for each group. In our molecular study, these two groups clearly resolved as being monophyletic within the *avidus*–*nigritarsis* lineage, with high bootstrap support for monophyly using both methodologies (MP = 100 and ML = 99).

The *clavipes* group includes four species previously described (*M.clavipes*, *M.quadrinotatus*, *M.vandergooti* and *M.velox*), as well as three species recognised herein. Two of those latter species are described based on newly discovered material held in different museum collections (*M.aenigmaticus* sp. nov. and *M.rufofemoris* sp. nov.). Discovery of the third species, *M.latens* sp. nov., is attributable to the integrative taxonomic approach we applied. Previous indications of the potential existence of divergent species on the Iberian Peninsula based on minor morphological differences among Iberian populations previously identified as *M.clavipes* are supported by our molecular and geometric morphometrics analyses. We also confirm the validity of *M.latens* sp. nov. as a new species through our combined morphological, molecular and geometric morphometrics analyses. Based on our analysis of the COI gene, *M.latens* sp. nov. is clearly different from the other two analysed species of the *clavipes* species group (i.e., *M.clavipes* and *M.velox*), as illustrated in both the Maximum-Parsimony and Maximum-Likelihood trees. This Iberian endemic displays a significantly different wing shape from *M.clavipes*, both in terms of species and population analyses. As revealed by many previous integrative hoverfly studies, wing shape is a reliable character for cryptic and sibling species delimitation. The strength of wing shape as a taxonomic character lies in its strong heritability (Moraes et al. 2004), with previous wing shape analyses proving concordant with molecular data (Vujić et al. 2013b, 2020a; Ačanski et al. 2016; Šašić et al. 2016; Radenković et al. 2018b; Kočiš Tubić et al. 2018; Chroni et al. 2018). Here, the high percentage of correct classification for specimens of *M.latens* sp. nov. and *M.clavipes* again validate that wing shape is a reliable diagnostic character for species assignment. Importantly, differences in the morphological characters used to formulate the key presented herein enable proper identification of all species from the *clavipes* group.

The *pruni* species group comprises two well-known species (*M.cupreus* and *M.pruni*), one new species (*Merodonaequalis* sp. nov.), and a previously described taxon, whose status has now been revised. Classically, *M.pallidus* was considered a synonym of *M.pruni*. This species was described based on the female holotype discovered during our research in the Paris Museum. Based on newly found specimens from different collections conspecific with the type, we characterised morphological traits that confirmed the status of *M.pallidus* stat. rev. as a valid independent taxon, redefined herein. *Merodonobscurus* was described as a variety of *M.pruni*, and synonymy with *M.pruni* was cited in recent literature. Based on the results of our morphological, molecular and geometric morphometrics analyses, *M.obscurus* stat. rev. represents an independent taxon distributed in North West Africa, i.e., far from the Eastern Mediterranean range of *M.pruni*. Moreover, our integrative taxonomic approach successfully resolved the taxonomic status of *M.obscurus*. Both our MP- and ML-based molecular analyses clearly resolved specimens of *M.obscurus* as a separate clade, with strong bootstrap support (99) distinguishing it from species *M.pruni*. Furthermore, our geometric morphometric analysis successfully separated *M.pruni* from *M.obscurus* based on wing shape, both in our species and population analyses. The accurate classification success rate for *M.obscurus* specimens further supports their distinctiveness, with only one specimen of *M.obscurus* being misclassified.

### ﻿Distribution

The two species groups we have examined herein, *clavipes* and *pruni*, have partially overlapping distributions. Both groups have diversified across the Mediterranean Basin. Several *Merodon* groups display similar patterns. For example, the *avidus* complex and the *natans* group are widespread in the Mediterranean Basin, but also have representatives on the Iberian Peninsula and in North Africa. Both those taxonomic clusters were the subject of recent integrative analyses and, as in our study, wing shape and molecular data successfully revealed their hidden diversity (Ačanski et al. 2016; Vujić et al. 2021c). Ačanski et al. (2016) deduced their diversification processes, likely a response to repeated isolation in parts of the Mediterranean Basin during glacial-interglacial cycles (Hewitt 1999, 2001; Konstantinov et al. 2009). Later, the Pyrenees probably acted as a geographical barrier to prevent dispersal of *M.obscurus* stat. rev. and *M.latens* sp. nov. to other European areas.

In the case of both species groups, nominal species (i.e., *Merodonclavipes* and *M.pruni*) display the most widespread distributions; – that of *clavipes* group stretches from France throughout most of central and southern Europe to Ukraine, south Russia and Turkey, while *pruni* group occupies most of southern Europe through to Ukraine and Turkey and extending further eastwards into Tajikistan and Pakistan. Looking closely at species distributional patterns, it is evident that the range of species in the *pruni* group is slightly more easterly than that of the *clavipes* group. In fact, only species *M.pruni* occurs in Europe, and all other species in the *pruni* group primarily occur in the Middle East and Central Asia. Furthermore, species *M.obscurus* stat. rev. occurs in North Africa, making it the only species in the two groups that is distributed here. *Merodoncupreus* and *M.aequalis* sp. nov. display the narrowest distributions of all species belonging to the *pruni* group, with *M.cupreus* only occurring in the eastern part of Turkey and *M.aequalis* sp. nov. being restricted to a few localities in Israel and the State of Palestine.

Regarding the *clavipes* group, the respective ranges of three out of its seven species include at least part of Europe. One of those three species (*M.latens* sp. nov.) is restricted to the Iberian Peninsula, whereas the other two (*M.clavipes* and *M.velox*) occur across central and southern Europe. The ranges of the other species in the *clavipes* group (*M.rufofemoris* sp. nov., *M.quadrinotatus* and *M.vandergooti*) are somewhat restricted to the Middle East and Central Asia. The distribution of *M.aenigmaticus* sp. nov. is puzzling, as the name suggests, but based on the distribution of its two closely related species (*Merodonrufofemoris* sp. nov. and *M.vandergooti*, distributed in Turkey and Iran), it is likely to be in the Middle East.

The fact that the distributions of the two species groups studied herein overlap in the Mediterranean Basin centres on the fact that this region represents one of the world’s 25 biodiversity hotspots (Myers et al. 2000). More specifically, this region serves as a centre of *Merodon* diversity (Vujić et al. 2012) probably due to its high diversity of bulbous plant species, which proved to be host plants for known larval stages (Andrić et al. 2014; Ricarte et al. 2017; Preradović et al. 2018). Unfortunately, host plant(s) for species of *pruni* group and *clavipes* group are still unknown and immature stages undescribed. Turkey displays the highest species diversity for both species groups assessed herein, hosting three of five *pruni*-group species and four of seven *clavipes*-group species, confirming its status as having the greatest diversity and endemicity of the genus *Merodon* in the Mediterranean Basin (Vujić et al. 2015). Although the Middle East and Central Asia appears to be less diverse and species-rich, greater research effort focused on these regions in recent years has highlighted the prevalence of *Merodon* species there (Vujić et al. 2013a; Vujić et al. 2019; Likov et al. 2020).

## ﻿Conclusions

Our revision of two closely-related *Merodon* species groups from the *avidus*–*nigritarsis* lineage, i.e., *pruni* and *clavipes*, uncovers four new species (*M.aenigmaticus* sp. nov., *M.aequalis* sp. nov., *M.latens* sp. nov. and *M.rufofemoris* sp. nov.) and confirms the status of six previously well-known species. In addition, we redescribe *M.pallidus* stat. rev., re-instating it as a valid species from synonymy with *Merodonpruni*. The integrative taxonomic approach we adopted again demonstrated its power in resolving hoverfly taxonomy. A combination of morphological, molecular and geometric morphometric analyses revealed the divergence between *M.latens* sp. nov. and *M.clavipes*, as well as *M.obscurus* stat. rev. and *M.pruni*. The two studied species groups display partially overlapping distributions, albeit with that of the *pruni* group being slightly more eastward relative to that of the *clavipes* group. The Anatolian Peninsula hosts three of the five *pruni*-group species and four of the seven *clavipes*-group species, representing the area with the highest *Merodon* diversity and endemicity across the Mediterranean Basin, Middle East and Central Asia.

## Supplementary Material

XML Treatment for Merodon aenigmaticus

XML Treatment for Merodon splendens

XML Treatment for Merodon latens

XML Treatment for Merodon quadrinotatus

XML Treatment for Merodon rufofemoris

XML Treatment for Merodon vandergooti

XML Treatment for Merodon aequalis

XML Treatment for Merodon cupreus

XML Treatment for Merodon obscurus

XML Treatment for Merodon pallidus

## ﻿Additional information

### Author ORCIDs

Ante Vujić https://orcid.org/0000-0002-8819-8079

Snežana Radenković https://orcid.org/0000-0002-7805-9614

Laura Likov https://orcid.org/0000-0002-7215-1006

Nataša Kočiš Tubić https://orcid.org/0000-0002-6077-7378

Grigory Popov https://orcid.org/0000-0002-2519-1937

Mihajla Djan https://orcid.org/0000-0002-2427-0676

Marina Janković Milosavljević https://orcid.org/0000-0002-2136-815X

Jelena Ačanski https://orcid.org/0000-0003-1745-6410

### Data availability

The data that support this study are available in the article and accompanying online Supplementary information. Nucleotide sequence data that support this study are available in GenBank at https://www.ncbi.nlm.nih.gov/genbank/.

## Supplementary materials

Supplementary material 1 List of molecular samples Data type xlsx Explanation note List of molecularly analysed samples with GenBank accession numbers (in boldface: newly generated sequences within this study). https://binary.pensoft.net/file/1057114This dataset is made available under the Open Database License (http://opendatacommons.org/licenses/odbl/1.0/). The Open Database License (ODbL) is a license agreement intended to allow users to freely share, modify, and use this Dataset while maintaining this same freedom for others, provided that the original source and author(s) are credited.Ante Vujić, Snežana Radenković, Laura Likov, Nataša Kočiš Tubić, Grigory Popov, Ebrahim Gilasian, Mihajla Djan, Marina Janković Milosavljević, Jelena Ačanski

Supplementary material 2 Material listed with occurences Data type xlsx Explanation note List of studied specimens, except type material cited in main document. https://binary.pensoft.net/file/1057115This dataset is made available under the Open Database License (http://opendatacommons.org/licenses/odbl/1.0/). The Open Database License (ODbL) is a license agreement intended to allow users to freely share, modify, and use this Dataset while maintaining this same freedom for others, provided that the original source and author(s) are credited.Ante Vujić, Snežana Radenković, Laura Likov, Nataša Kočiš Tubić, Grigory Popov, Ebrahim Gilasian, Mihajla Djan, Marina Janković Milosavljević, Jelena Ačanski

Supplementary material 3 DNA data Data type pdf Explanation note Maximum-Likelihood tree based on the General Time Reversible model. The tree with the highest log likelihood (-8297.00) is shown. A discrete Gamma distribution was used to model evolutionary rate differences among sites (5 categories (+G, parameter = 1.1113)). The rate variation model allowed for some sites to be evolutionarily invariable ([+I], 59.56% sites). Bootstrap values ≥ 50 are depicted near nodes. https://binary.pensoft.net/file/1057116This dataset is made available under the Open Database License (http://opendatacommons.org/licenses/odbl/1.0/). The Open Database License (ODbL) is a license agreement intended to allow users to freely share, modify, and use this Dataset while maintaining this same freedom for others, provided that the original source and author(s) are credited.Ante Vujić, Snežana Radenković, Laura Likov, Nataša Kočiš Tubić, Grigory Popov, Ebrahim Gilasian, Mihajla Djan, Marina Janković Milosavljević, Jelena Ačanski
