# Supplementary figures and images for: ﻿Revisions of the clavipes and pruni species groups of the genus Merodon Meigen, 1803 (Diptera, Syrphidae)
Source: Zookeys. 2024 May 28;1203:1–69. doi: 10.3897/zookeys.1203.118842 (PMC11150873; doi:10.3897/zookeys.1203.118842)

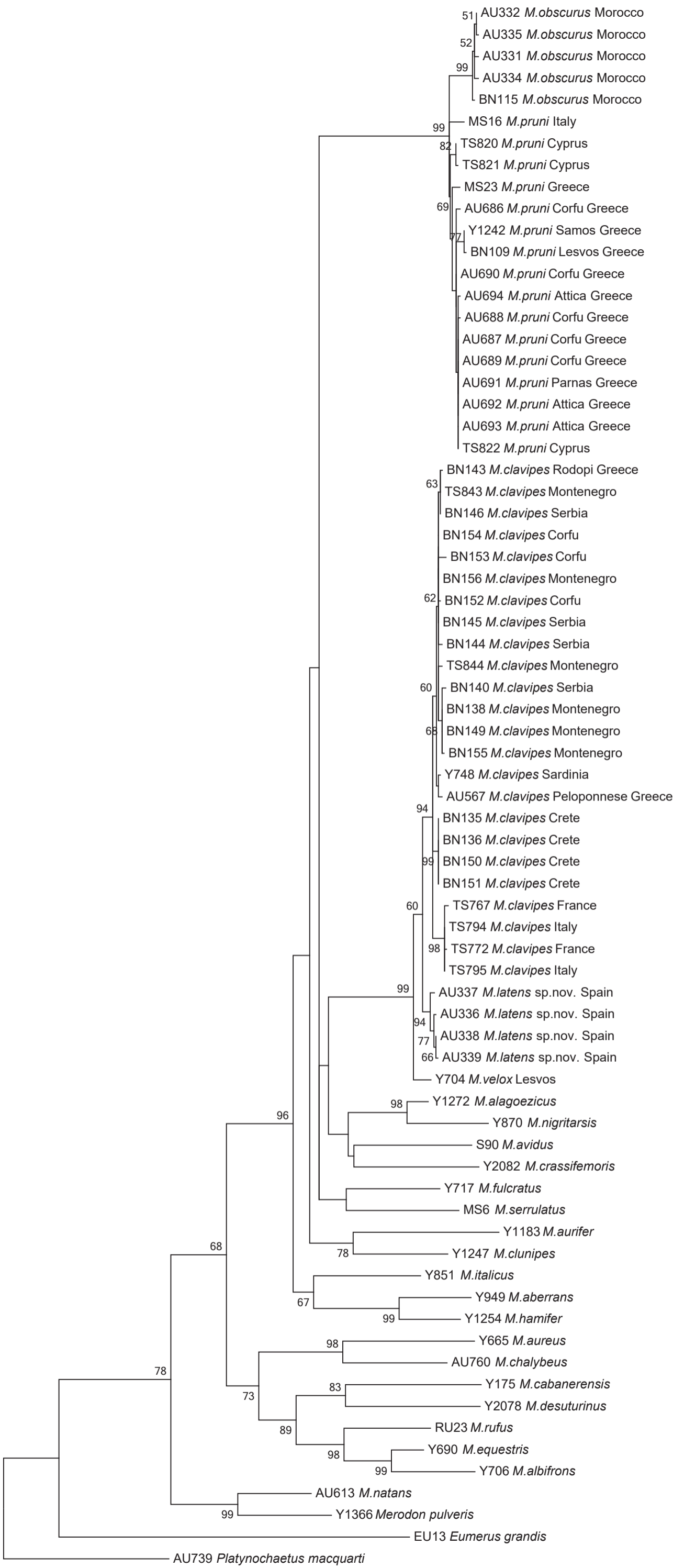

0.02

Supplement: Supplementary material 3 — DNA data [file zookeys-1203-001_article-118842__-s003.pdf]
